# Determination of dosage compensation and comparison of gene expression in a triploid hybrid fish

**DOI:** 10.1186/s12864-016-3424-5

**Published:** 2017-01-05

**Authors:** Li Ren, Chenchen Tang, Wuhui Li, Jialin Cui, Xingjun Tan, Yafeng Xiong, Jie Chen, Jun Wang, Jun Xiao, Yi Zhou, Jing Wang, Min Tao, Chun Zhang, Shaojun Liu

**Affiliations:** State Key Laboratory of Developmental Biology of Freshwater Fish, Hunan Normal University, Changsha, 410081 China

**Keywords:** Dosage compensation, Genomic dominance, Biased expression, Triploid, Transcriptome

## Abstract

**Background:**

Polyploidy and hybridization are both recognized as major forces in evolution. Most of our current knowledge about differences in gene regulation in polyploid hybrids comes from plant studies. The gene expression of diverged genomes and regulatory interactions are still unclear in lower vertebrates.

**Results:**

We generated 229 million cleaned reads (42.23 Gbp) from triploid of maternal grass carp (*Ctenopharyngodon idellus*, Cyprininae, 2n = 48) × paternal blunt snout bream (*Megalobrama amblycephala*, Cultrinae, 2n = 48) and their diploid parents using next-generation sequencing. In total, 157,878 contigs were assembled and 15,444 genes were annotated. We examined gene expression level changes among the parents and their triploid offspring. The mechanisms of dosage compensation that reduced triploid expression levels to the diploid state were determined in triploid fish. In this situation, novel gene expression and gene silencing were observed. Then, we established a model to determine the extent and direction of expression level dominance (ELD) and homoeolog expression bias (HEB) based on the relative expression level among the parents and their triploid offspring.

**Conclusions:**

Our results showed that the genome-wide ELD was biased toward maternal genome in triploid. Extensive alterations in homoeolog expression suggested a combination of regulatory and epigenetic interactions through the transcriptome network. Additionally, the expression patterns of growth genes provided insights into the relationship between the characteristics of growth and underlying mechanisms in triploids. Regulation patterns of triploid state suggest that various expression levels from the initial genomic merger have important roles in adaptation.

**Electronic supplementary material:**

The online version of this article (doi:10.1186/s12864-016-3424-5) contains supplementary material, which is available to authorized users.

## Background

Polyploid hybrids that play a role in the origin of plant and animal species have been studied for many years. Hybridization is viewed as a destructive process that counteracts speciation and delays evolution [1]. However, biologists increasingly find new examples where hybridization seemed to facilitate speciation and adaptive radiation in animals and plants [2]. Although polyploidy and hybridization can be viewed separately, the processes often occur together in the form of allopolyploidy [3]. Allotriploidy is rarely discovered in lower vertebrates except of triploid edible frog *Rana esculenta* [4], the triploid cyprinidae fish of *Squalius alburnoides* complex [5], the triploid of *Ctenopharyngodon idellus* × *Megalobrama amblycephala*, the triploid of *Carassius auratus* red var. and *Cyprinus carpio* [6, 7]. The coexistence of divergent parental genomes begins with heterozygosity and heterosis in F_1_ hybrids [2], whereas gene redundancy shields hybrids from the deleterious effects of mutations [2, 8].

The molecular mechanisms of gene expression regulation in allotetraploids are well studied in plants. However, only a few animal species, mostly insects and fish, have been recognized as being the result of hybridization and polyploidy [9]. Therefore, little has been done to understand the effects of ploidy increases on gene regulation and their impact on the evolutionary potential of populations. Both the *Squalius alburnoides* complex and triploid Chinook salmon are appropriate systems to research gene copy silencing that is attributed to complex dosage-compensation mechanisms [5, 9–11]. Although the responsible molecular mechanisms have not been determined, some hypotheses have been proposed to explain this fundamental biological phenomenon. In cyprinid fishes, a few reports described the dosage effect of the house-keeping gene *β-actin* between triploids and diploids, in which the absolute expression level was estimated to be 1:1 [12]. This gene could be used as an internal control in the study of mRNA and microRNA expression levels in triploids [12–15]. Additionally, the dosage effect of functional genes including growth-hormone was detected in triploid salmon [16]. Although triploids also exhibited higher narrow-sense heritability values relative to diploid salmon, maternal effects were estimated to be generally lower in triploids than in diploids. The dosage effects resulting from adding an extra set of chromosomes to maternal genome are primarily additive [17].

Compared with either parent, a stable and distinct hybrid will result from hybridization if reproductive isolation is weak. Therefore, hybrid species usually are considered as a third cluster of genotypes [18]. However, evolution normally occurs by small adjustments rather than saltation. The expression pattern of homologous genes is the focus of our attention. Recent reports show that duplicate gene pairs in hybrids may display homoeolog expression bias (HEB), where the two homoeologs are expressed unequally and often vary among tissues [19, 20]. The epigenetic remodeling including nuclear enlargement and increased complexity of the processes during cell division always results in both the activation and suppression of gene expression in polyploids [2]. In addition to HEB, a second phenomenon was more recently described: expression silencing of parental homoeologs and the formation of novel genes are some of the consequences that the new polyploid genome may experience [21, 22]. Different from genome diploidization in autotetraploids, the merge of the A and D genome in hybrids often resulted in a variety of expression regulation changes that occurred in either parental homoeolog, and the differential homoeolog expression and homoeologs silencing patterns were reported in allopolyploid cotton and fungi [23, 24].

Molecular mechanisms, or even the specific biological processes that are involved with changes in gene expression levels in polyploids, are largely unknown. Differences in growth and survival commonly are observed in early stages in allopolyploids. Triploids of *Ctenopharyngodon idellus* × *Megalobrama amblycephala* are reported to have significantly higher growth rates than their diploid parents [6]. Hybrid growth disorders always refer to the decreased growth or overgrowth that is identified in hybrid individuals. A study of hybrid mice that investigated the possible causes for hybrid growth disorders revealed that gene imprinting had a major effect [25]. Hybrid growth disorders may also be known as growth dysplasia [26]. At the same time, the increased amount of DNA may result in the larger cell volume of polyploids relative to their diploid progenitors [27, 28]. However, comparisons of inbred diploid and polyploid salamanders [29] and mice [30] indicate that the larger cells in polyploids did not necessarily result in larger bodies. Instead, a developmental mechanism regulates organ growth to compensate for cell size. Another hypothesis supports the idea that the larger cells in polyploids were attributed to high metabolic rates and result in high growth rates [31]. After triploidization, the change in growth function in triploids would be determined by various of growth regulation mechanisms.

In this study, we investigated the liver transcriptome in diploid parents (*Ctenopharyngodon idellus*, ♀ × *Megalobrama amblycephala*, ♂) and their triploid offspring. The three sets of chromosomes allowed us to analyze the global expression level in triploid. Compared to the expression level of the diploid parents, we detected a negative dosage effect in triploids. Then, the genomic constitution of two sets of maternal homoeologs and one set of paternal homoeologs allowed us to investigate the expression pattern in triploid offspring. We characterized gene expression patterns according to the 12 possible categories, including mid-parents, up- and down-parent, maternal-dominance, and paternal-dominance [22, 32]. The aim of this study was to assess the magnitude and directionality of ELD and HEB in triploids. Furthermore, we detected the expression patterns in growth-related genes in triploid offspring and the inbred parents, and we discussed their relationship with the characteristic of rapid growth. Therefore, these results provide a novel perspective to describe expression regulation in triploids and hint at the underlying mechanism of triploidy.

## Results

### Transcriptome assembly

**Fig. 1:**
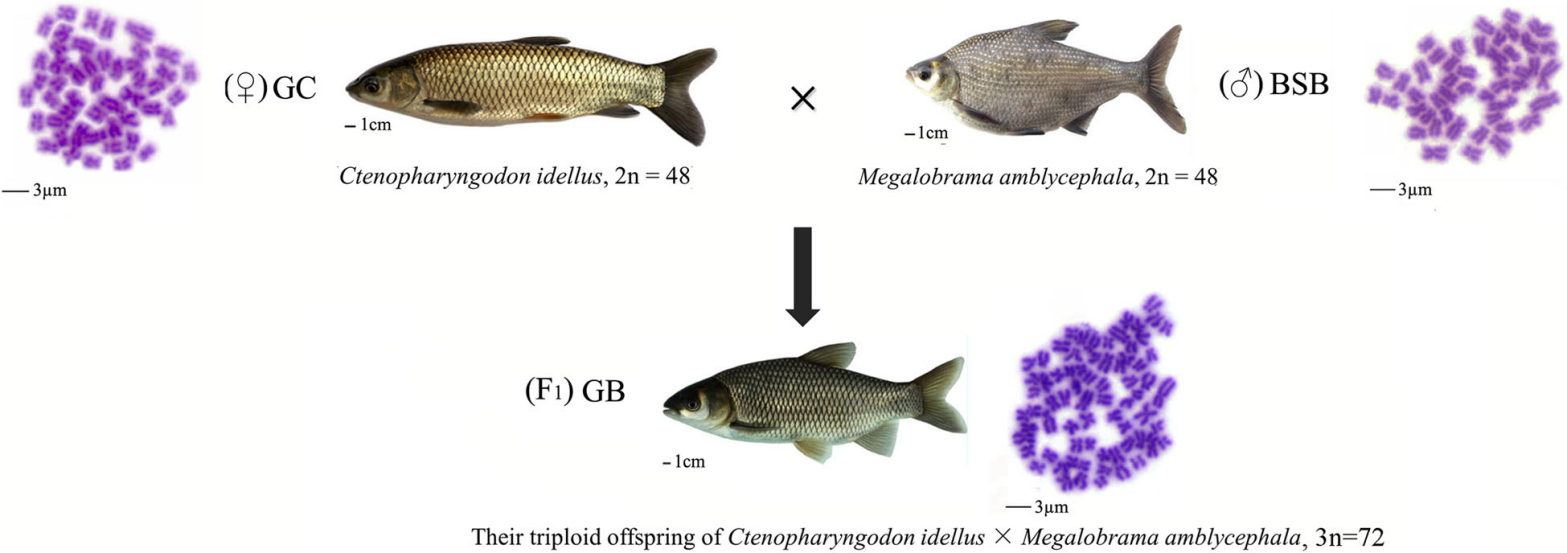
The chromosomal trait and appearance of grass carp (maternal GC = 48), blunt snout bream (paternal BSB = 48) and their triploid offspring (GB = 72)

To examine the changes in the global transcriptomic profile in triploid of *Ctenopharyngodon idellus* and *Megalobrama amblycephala* (GB), we obtained nine liver transcriptomes from maternal *Ctenopharyngodon idellus* (GC), paternal *Megalobrama amblycephalae* (BSB), and triploid offspring GB (Fig. 1).

The paired-end sequencing (PE × 90) had performed based on the nine libraries of the two parents and their triploid offspring. The basic information was summarized in Table 1. After the initial adapter trimming and quality filtering, we had collected all 299.03 million cleaned reads from the nine libraries (Table 1). Then, we assembled the 100.14 (BSB), 96.77 (GC) and 102.12 (GB) million cleaned reads (42.23 Gb) using Trinity, separately. Among of 157,878 assembled contigs in three species, the number of contigs (≥1000 bp) were 11,190 in paternal BSB, 9,873 in maternal GC, and 11,005 in triploid GB (Table 1).

**Table 1:** Summary of obtained transcriptome data

	BSB-1	BSB-2	BSB-3	GC-1	GC-2	GC-3	GB-1	GB-2	GB-3	Total	Merge-sequences
Total reads (million)	55.29	23.69	21.16	51.60	23.52	21.65	55.33	23.63	23.16	299.03	
Total nucleotides (Gb)	4.98	4.79	4.27	4.64	4.75	4.37	4.98	4.77	4.68	42.23	
Total length (bp)	40,397,155			36,773,931			40,344,526			117,515,612	77,813,385
N50 length (bp)	1,499			1,181			1,330				1,616
Mean length (bp)	788.70			710.65			734.73				813.08
NO. assembled sequences (≥100 bp)	51,220			51,747			54,911			157,878	95,702
NO. assembled sequences (≥1000 bp)	11,190 (21.85%)			9,873 (19.08%)			11,005 (20.04%)			32,068	
Q20 percentage (%)	97.1	93.88	93.78	97.9	93.57	92.49	97.04	92.35	93.16		
GC percentage (%)	48.23	48.69	48.68	48.42	50.29	50.41	47.65	48.58	48.78		

### Functional analyses

Using BLASTX (e-value ≤ 1e^−6^) against NCBI-NR, Swiss-Prot, Kyoto Encyclopedia of Genes and Genomes (KEGG), Clusters of Orthologous Groups (COG) and Gene Ontology (GO) databases (alignment length ≥100 bp), 28,950 sequences from paternal BSB, 29,110 sequences from maternal GC, and 29,255 sequences from triploid GB were identified as annotated sequences. The sequence distribution of annotated sequences in the above five public databases and the e-value distribution of annotated genes are shown in Additional file 1. After BLASTX alignment, we performed GO analysis (level 2). The distribution of gene annotations showed the function differences between the parents and their hybrids (Additional file 2). To obtain more accurate information about the gene expression in the three species, our next analysis was focused on the 13,893 shared genes (Additional file 3).

### Differential expression between diploid and triploid species

To investigate expression level in the two diploid parents and their triploid offsprings, a total of 157,878 contigs from nine individuals were clustered by CD-HIT, and the 95,702 reference transcript contigs were obtained from clustering (Additional file 4). Then, the total reads from the nine samples were mapped to the 95,702 reference transcripts using BLAST-like alignment tool (Blat) (Additional file 5) [33]. According to the mapping results, we detected the silenced genes (GB = 0, GC > 10, and BSB > 10) and novel genes based on the read counts (GB > 10, GC = 0, and BSB = 0) in triploid offspring, the 27 genes appeared to be silenced, and two genes exhibited a novel expression pattern (Additional file 6).

**Fig. 2:**
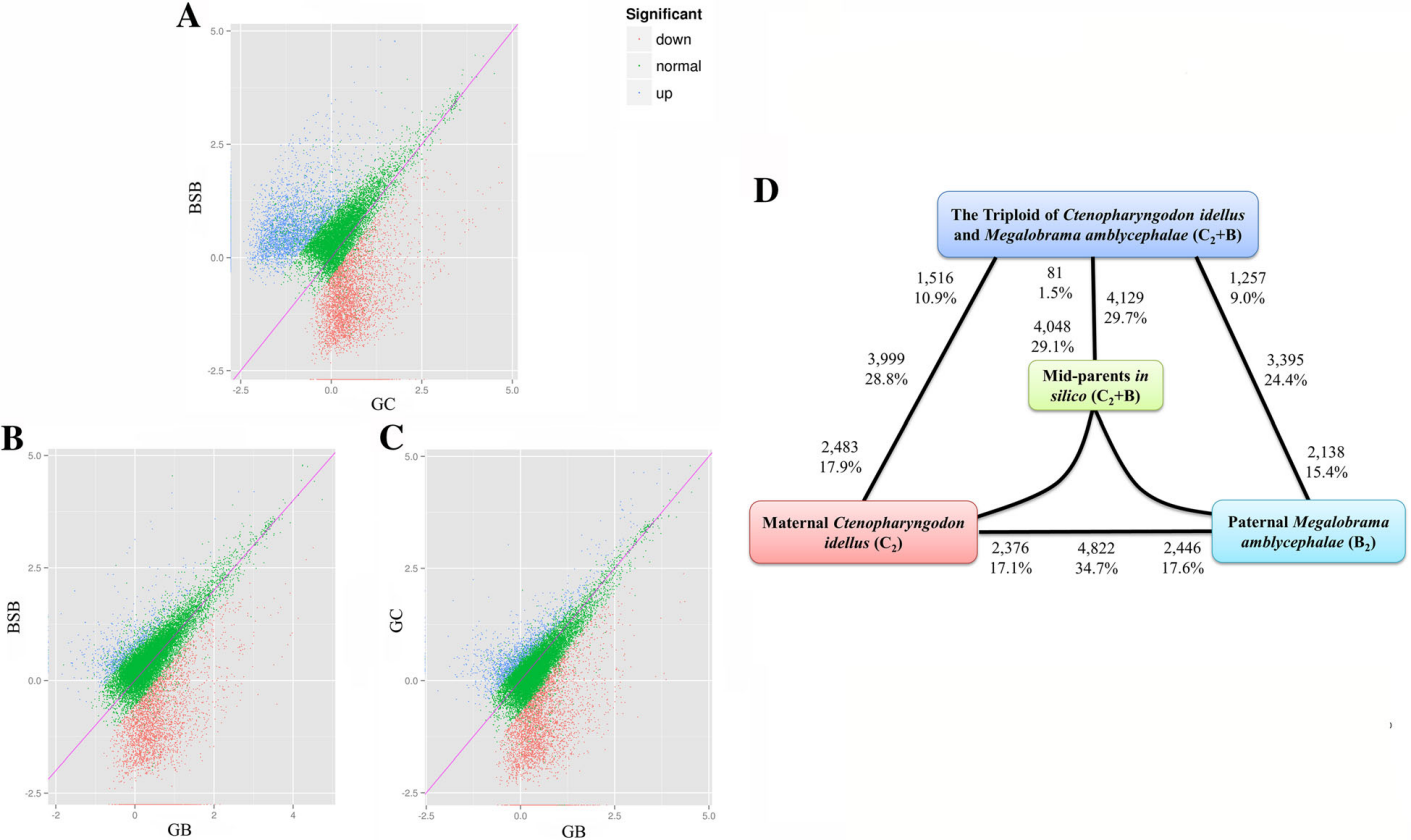
Differentially expressed genes in maternal GC, paternal BSB, and triploid offsprings GB. **a**. The different expression level between BSB and GC is shown. **b**. The different expression level between BSB and GB is shown. **c**. The different expression level between GC and GB is shown. The red (blue) points in the graph (MA-plot) are the genes that were identified as differentially expressed. The green points in the graph (MA-plot) are the genes that were not significantly different. Differentially expressed genes were identified using MA plot-based methods with a random sampling model and a p-value threshold of 0.001. **d**. Differentially expressed genes in each contrast between triploid offspring and their origin parents. Bold text exhibits the total number and fraction of genes differentially expressed in each contrast. Also shown for each contrast is the partitioning of the total number of differentially expressed genes into the direction of upregulation. For example, 4,822 genes are indicated as being differentially expressed between *M. amblycephala* and *C. idellus*. Of these, 2,376 are upregulated in *C. idellus*, and 2,446 genes are upregulated in *M. amblycephala*

To detect significant differentially expression, false discovery rate (FDR) < 0.001 and the absolute value of log_2_ ratio > 1 were used as thresholds in comparison of the two parents and their triploid offsprings. In all comparisons, the percentage of genes showing differential expression between the F_1_ triploids and the two parents was asymmetric (*P* < 0.05; Fisher’s exact test). Comparison of the expression level in the two parents revealed that 2,446 genes were up-regulated in paternal BSB, and 2,376 genes were up-regulated in maternal GC (Fig. 2a and d). We compared the gene expression in paternal BSB and triploid GB, and we determined that 2,138 genes were up-regulated in BSB, and 1,257 genes were up-regulated in GB (Fig. 2b and d). Then, we compared the expression of maternal GC and triploid GB; 2,483 genes were up-regulated in GC, and 1,516 genes were up-regulated in GB (Fig. 2c and d).

**Fig. 3:**
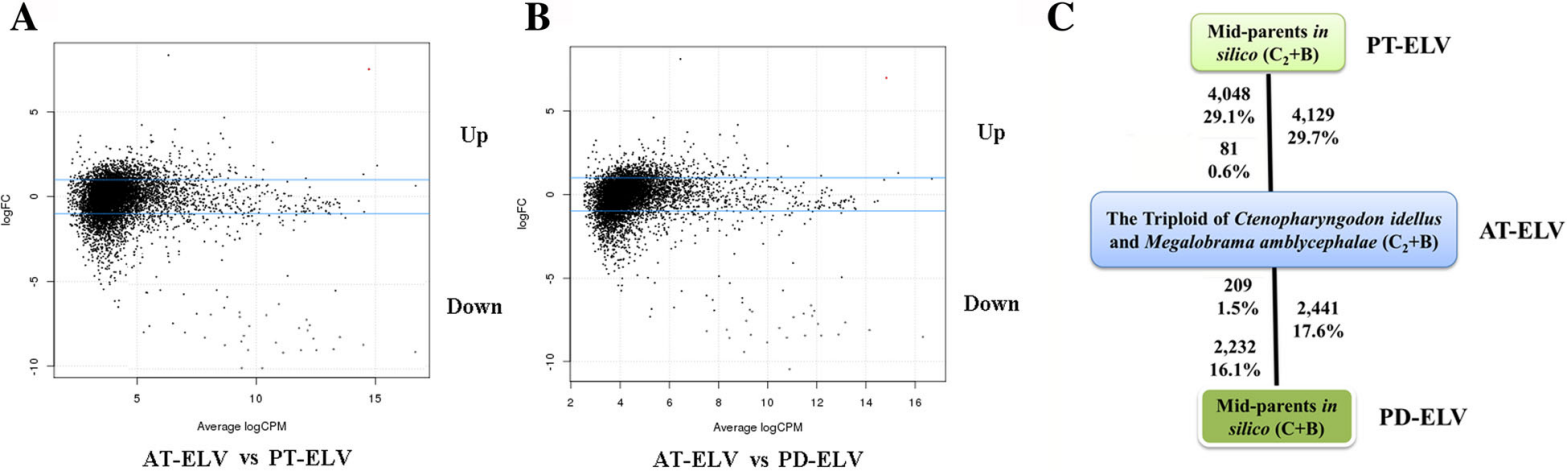
Distribution of differentially expressed genes as compared AT-ELV with PT-ELV (**a**) and compared AT-ELV with PD-ELV (**b**) in MA plot. **a**. Compared AT-ELV and PT-ELV, Black dots between the two blue line represents the genes with no significant difference and others exhibit as significantly differential expression (>2-fold change and FDR < 0.05), respectively. **b**. Compared AT-ELV and PD-ELV, Black dots between the two blue line represents the genes with no significant difference and others exhibit as significantly differential expression (>2-fold change and FDR < 0.05), respectively. **c**. Bold text exhibits the total number and fraction of genes differentially expressed between the expressions of triploid offspring with the predicted expression of mid-parents *in silico* module of C_2_ + B and C + B

To detect whether the phenomenon of dosage effect occurred in triploidstriploid, the comparison of the value of predicted triploid expression level (PT-ELV, also known as *in silico* mid-parents C_2_ + B) and the value of actual triploid expression level (AT-ELV) of GB was performed (see methods). The 4,048 genes (29.1%) had exhibited up-regulated expression in PT-ELV of GB and only 81 genes (0.6%) had shown up-regulated expression in AT-ELV of GB (Fig. 3a and c). The above results were obviously showing that the negative dosage effect of maternal GC-homoeologous chromosomes had occurred in triploid offspring. Based on the existence of dosage effect, we had hypothesized the value of predicted diploid expression level (PD-ELV, also known as *in silico* mid-parents C + B) and compared it with the AT-ELV. The 2,441 genes that were significantly differentially expressed in triploids included 2,232 (16.1%) up-regulated genes in PD-ELV of GB and 209 (1.5%) up-regulated genes in AT-ELV of GB (Fig. 3b and c). Our results shed insight into that both the mechanism of negative dosage effects and another unknown mechanism result in triploid expression level decreasing to the diploid state.

### Expression patterns under dosage effect

**Fig. 4:**
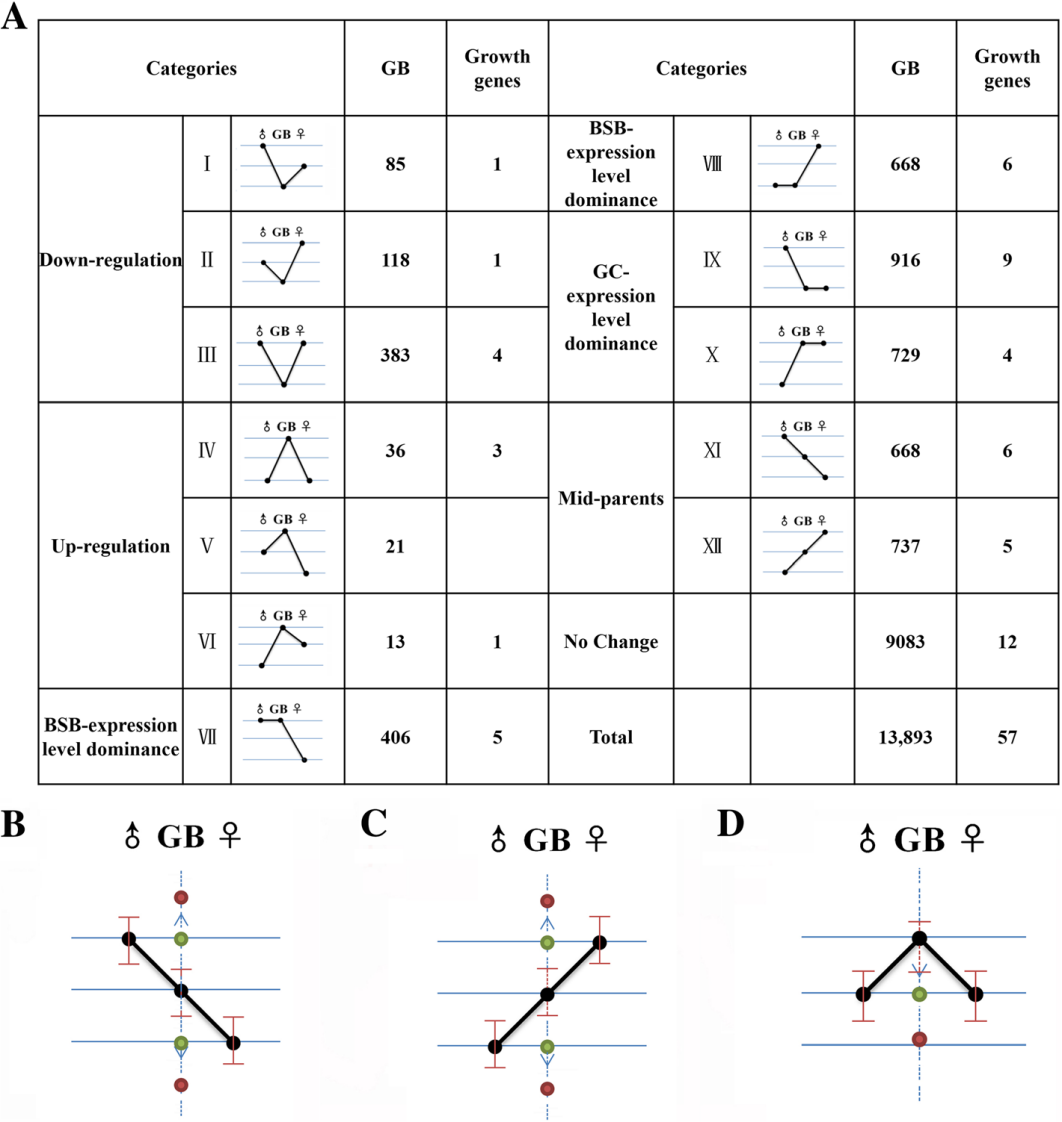
Partitioning of expression patterns in triploid. **a**. The 12 possible differential expression states in triploid. Roman numerals indicate the same categories that were used in Rappet et al*.* (2009) [32]. The respective gene expression patterns for the diploid parents and their triploid offspring are shown in the schematic graphs. **b**. GB expression levels (*black* spot in the dotted line) when paternal BSB (♂) has higher expression than maternal GC (♀). The significantly different expression levels in triploid that was lower than those in paternal BSB and higher than those in maternal GC show the mid-parents expression pattern (XI). If, however, the GB expression was not significantly different (threshold value of log_2_Ratio ≤ 1) from that of the parents (*green* spots), the ELD in the direction of paternal BSB or maternal GC can be explained by up- or downregulation of the BSB homoeolog (VII and IX). The significantly different expression levels in which triploid expression was higher than that of paternal BSB or maternal GC (*red* spot above the midpoint) or lower than that of paternal BSB or maternal GC (*red* spot below the midpoint) conformed to the upregulation (V) and downregulation (I) patterns, respectively. **c**. GB expression levels (*black* spot in the dotted line) if maternal GC (♀) has higher expression than paternal BSB (♂). **d**. GB expression levels (*black* spot in the dotted line) if the expression levels of paternal BSB or maternal GC were not significantly different

As a prerequisite of the dosage effect found in triploid, it shed us insight into the expression level raised from one paternal set of chromosomes and one maternal set of chromosomes in triploid. For better understanding ELD and HEB under dosage effects, we had established 12 categories including mid-parents (XI and XII), up/down expression (I, II, III, IV, V, and VI), and ELD (VII, VIII, IX and X) to assess differential gene expression (see Methods). Among of 13,893 shared genes, 2,749 genes (19.8%) were detected as ELD category (Fig. 4a). Maternal GC-ELD including 1,645 genes (11.8% of all genes, categories IX and X) had exhibited more influence than paternal BSB-ELD (1,104 genes, 7.9% of all genes, categories VII and VIII) in triploid (Fig. 4a). Categories VII and X (GC vs BSB = 1.8 vs 1) represented the up-regulated ELD, while down-regulated ELD (GC vs BSB = 1.3 vs 1) was detected in categories VIII and IX in triploid (Fig. 4a). The results showed that the number of HEB genes was unbalanced in triploid with respect to the original parent was inclined to maternal GC genome (paternal BSB bias vs maternal GC bias = 1,104 vs 1,645) (Fig. 4a). To compare triploid GB with paternal BSB, we examined the 1,536 up-regulated genes (IV, V, VI, X, and XII) and 2,170 down-regulated genes (I, II, III, IX, and XI). Compared with maternal GC, the 1,144 up-regulated genes (IV, V, VI, VII, and XI) and 2,021 down-regulated genes (I, II, III, VIII, and XII) was examined in triploid (Fig. 4a). The gene number related to down- or up-regulation had a global mRNA preference toward down-regulation (up-regulation vs down-regulation = 70 vs 586). In addition, 65.4% (9,083 genes, categories of no changes) showed similar expression levels in the parents.

### The expression level of growth genes in the hybrid

To analyze the expression level using the 12-categories model, comparison of GB with both of parents indicated that hybridization and triploidization not only resulted in the up-regulation of some genes (70 genes, 0.6%, categories IV-VI) but also lead to the down-regulation in a large number of genes (586 genes, 29.1%, categories I-III). To study on function of growth-regulated in triploid, we obtained 57 shared growth genes among triploid offspring and their parents in the following analysis (Fig. 4a). Analysis of the differential expression of growth-related genes among the shared growth genes revealed that 7.0% (4 genes, categories IV-VI) of genes were up-regulated and 10.5% (10 genes, categories I-III) of genes were down-regulated (Table 2, Fig. 4a). The ratio of the number of up-regulated genes in the growth function category was higher than the total ratio of up-regulated genes (*P* < 0.05; Fisher’s exact test).

**Table 2:** Basic information of differential expression growth genes among the triploid hybrids and its parents

Categories	Ensembl protein id	BSB-RPKM	GB-RPKM	GC-RPKM	Symbols	Go term name	Go term accession
IX	ENSDARP00000003167	1.28	0.05	0.09	*cds2*	positive regulation of vascular endothelial growth factor signaling pathway	GO:1900748
XI	ENSDARP00000074846	10.44	4.68	2.00	*foxj2*	vascular endothelial growth factor receptor signaling pathway, regulation of organ growth	GO:0048010, GO:0046620
XI	ENSDARP00000013686	4.94	1.97	0.59	*bmp2a*	growth, growth factor activity	GO:0040007, GO:0008083
III	ENSDARP00000072198	6.95	1.36	3.51	*bambia*	transforming growth factor beta-activated receptor activity	GO:0005024
IV	ENSDARP00000023951	2.24	8.00	3.67	*insra*	developmental growth	GO:0048589
IX	ENSDARP00000102655	2.97	1.31	1.07	*sall1a*	positive regulation of fibroblast growth factor receptor signaling pathway	GO:0045743
I	ENSDARP00000011459	2.40	0.16	0.42	*ppm1bb*	negative regulation of transforming growth factor beta receptor signaling pathway	GO:0030512
IX	ENSDARP00000003654	9.23	0.88	0.57	*fgf13b*	growth factor activity	GO:0008083
XII	ENSDARP00000106837	0.15	1.88	5.17	*suv420h2*	regulation of multicellular organism growth	GO:0040014
XII	ENSDARP00000111890	0.62	2.54	7.51	*cyr61l2*	regulation of cell growth, insulin-like growth factor binding	GO:0001558, GO:0005520
VIII	ENSDARP00000089039	2.43	1.83	7.18	*nrp1b*	vascular endothelial growth factor receptor signaling pathway, vascular endothelial growth factor signaling pathway, regulation of vascular endothelial growth factor receptor signaling pathway, growth factor binding, vascular endothelial growth factor-activated receptor activity	GO:0048010, GO:0038084, GO:0030947, GO:0019838, GO:0005021
VIII	ENSDARP00000091440	2.78	2.76	7.82	*skia*	negative regulation of transforming growth factor beta receptor signaling pathway	GO:0030512
XII	ENSDARP00000110575	0.48	1.19	5.87	*vegfaa*	vascular endothelial growth factor receptor signaling pathway, growth factor activity	GO:0048010 GO:0008083
VII	ENSDARP00000050533	3.95	2.63	0.71	*crim1*	regulation of cell growth, insulin-like growth factor binding	GO:0001558 GO:0005520
XI	ENSDARP00000028652	17.97	7.00	2.72	*pdgfrb*	platelet-derived growth factor receptor signaling pathway, platelet-derived growth factor alpha-receptor activity	GO:0048008 GO:0005018
XI	ENSDARP00000053094	3.57	1.61	0.63	*rhbdf1*	regulation of epidermal growth factor receptor signaling pathway, growth factor binding	GO:0042058 GO:0019838
III	ENSDARP00000030150	4.56	1.24	6.39	*smad1*	transforming growth factor beta receptor signaling pathway	GO:0007179
XI	ENSDARP00000052422	5.17	1.31	0.49	*spry2*	negative regulation of fibroblast growth factor receptor signaling pathway	GO:0040037
VII	ENSDARP00000036006	3.67	2.64	0.89	*gna13b*	unidimensional cell growth	GO:0009826
IX	ENSDARP00000071913	3.91	1.09	1.44	*si:dkey-101 k6.5*	transforming growth factor beta receptor signaling pathway, transforming growth factor beta receptor activity, type II	GO:0007179 GO:0005026
III	ENSDARP00000031108	6.34	1.10	3.85	*smad9*	transforming growth factor beta receptor signaling pathway	GO:0007179
XII	ENSDARP00000002466	0.20	2.85	6.13	*flt1*	vascular endothelial growth factor receptor signaling pathway, vascular endothelial growth factor-activated receptor activity	GO:0048010 GO:0005021
IX	ENSDARP00000032226	8.98	3.64	3.80	*extl3*	positive regulation of fibroblast growth factor receptor signaling pathway	GO:0045743
X	ENSDARP00000007058	1.31	3.00	4.18	*gpc4*	unidimensional cell growth	GO:0009826
IV	ENSDARP00000019643	23.24	54.31	25.38	*igfbp2b*	regulation of cell growth, insulin-like growth factor binding	GO:0001558 GO:0005520
VIII	ENSDARP00000039831	2.31	2.47	6.37	*tgfbr2*	transforming growth factor beta receptor signaling pathway, transforming growth factor beta receptor activity, type II	GO:0007179 GO:0005026
VI	ENSDARP00000057368	0.02	4.36	2.07	*igfbp5a*	regulation of cell growth, insulin-like growth factor binding	GO:0001558 GO:0005520
IX	ENSDARP00000060763	8.41	2.71	2.02	*spred1*	regulation of vascular endothelial growth factor receptor signaling pathway	GO:0030947
VII	ENSDARP00000076657	5.77	5.22	1.11	*gdf2*	growth, growth factor activity	GO:0040007 GO:0008083
IX	ENSDARP00000076455	5.53	1.00	1.56	*fgf19*	growth factor activity	GO:0008083
XII	ENSDARP00000018513	0.32	1.72	7.99	*mdkb*	growth factor activity	GO:0008083
IX	ENSDARP00000096601	10.28	3.34	2.55	*insrb*	developmental growth	GO:0048589
VII	ENSDARP00000047568	30.01	25.43	11.67	*igf2b*	forebrain development, fin regeneration, insulin-like growth factor receptor binding, growth factor activity	GO:0005159 GO:0008083 GO:0030900 GO:0031101
IX	ENSDARP00000025519	5.09	1.37	1.12	*tgfb3*	growth, transforming growth factor beta receptor signaling pathway, cell growth, growth factor activity, transforming growth factor beta receptor binding	GO:0040007 GO:0007179 GO:0016049 GO:0008083 GO:0005160
II	ENSDARP00000056452	0.06	0.01	17.17	*igfbp1b*	regulation of cell growth, insulin-like growth factor binding, insulin-like growth factor I binding, insulin-like growth factor II binding	GO:0001558 GO:0005520 GO:0031994 GO:0031995
VIII	ENSDARP00000058288	2.73	3.92	8.08	*dusp22b*	transforming growth factor beta receptor signaling pathway	GO:0007179
X	ENSDARP00000076869	1.53	3.92	4.53	*tgif1*	regulation of transforming growth factor beta receptor signaling pathway	GO:0017015
VIII	ENSDARP00000103673	1.63	2.82	8.90	*rabep1*	growth factor activity	GO:0008083
IV	ENSDARP00000017449	21.24	53.65	19.72	*igf1*	growth factor activity, insulin-like growth factor receptor binding	GO:0008083 GO:0005159
XI	ENSDARP00000032935	3.94	1.69	0.73	*ctnnb1*	positive regulation of fibroblast growth factor receptor signaling pathway	GO:0045743
III	ENSDARP00000075360	5.09	2.16	6.13	*fgfr2*	fibroblast growth factor receptor signaling pathway, fibroblast growth factor-activated receptor activity	GO:0008543 GO:0005007
VIII	ENSDARP00000008469	1.43	1.84	7.07	*bmpr2b*	transforming growth factor beta-activated receptor activity	GO:0005024
X	ENSDARP00000014981	1.46	3.34	6.61	*pink1*	regulation of vascular endothelial growth factor signaling pathway	GO:1900746
VII	ENSDARP00000069999	0.67	0.65	0.09	*gdf6a*	growth, growth factor activity	GO:0040007 GO:0008083
X	ENSDARP00000024277	5.83	18.91	19.20	*smad7*	transforming growth factor beta receptor signaling pathway	GO:0007179

After detecting the ELD of growth-regulated genes in triploid, eleven genes exhibited a paternal BSB-ELD, and 13 genes were showed a maternal GC-ELD (Fig. 4a). The percent of maternal GC-ELD (22.8%) of growth genes was higher than that of the total genes (11.8%). The percent of paternal BSB-ELD (19.3%) of growth genes was higher than that of the total genes (8.9%). The percent of parent ELD in growth-related genes was more than other genes in triploid. Eleven genes were considered to be mid-parent genes, and the remaining 12 growth-related genes showed no change in expression levels (Fig. 4a). The 21.1% of growth-related genes in the “No Change” category was lower than the 65.4% of total genes in that category (Additional file 7). These results suggest that there are more changes in growth-related gene expression in triploid than in other gene functions.

### Real-time quantitative PCR (qPCR) validation

**Fig. 5:**
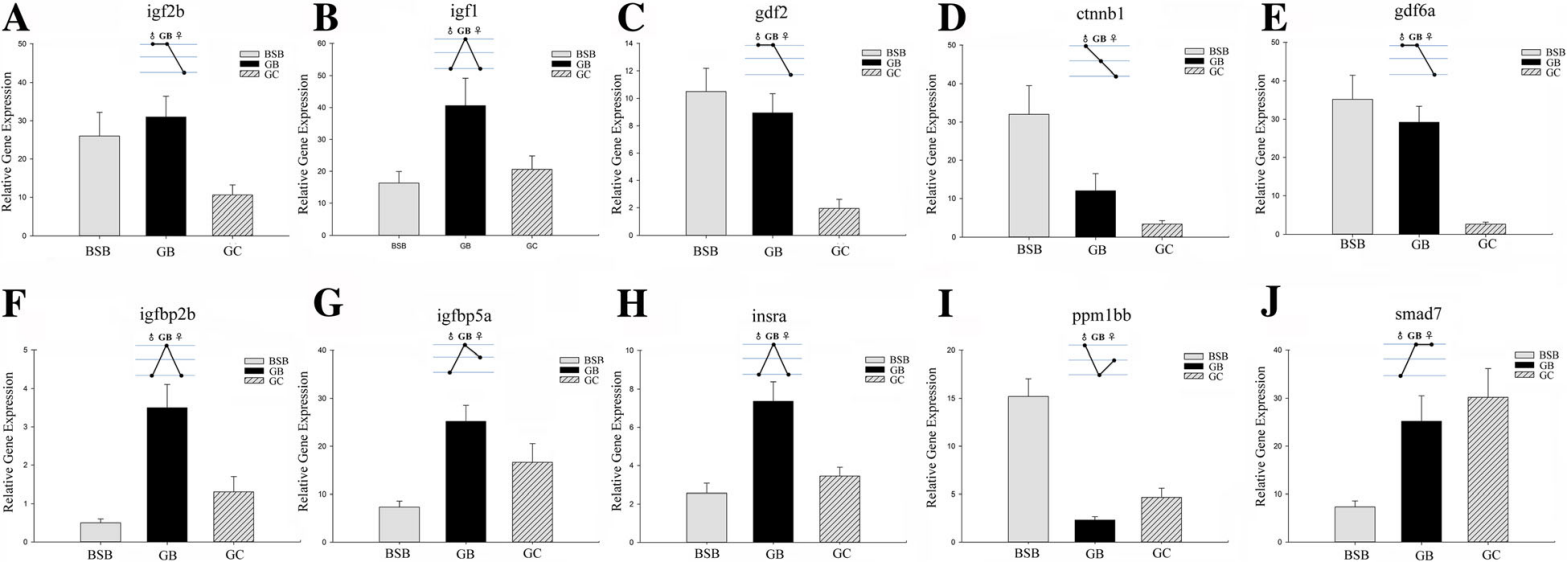
Real-time PCR analysis for ten differentially expressed genes: **a**. *igf2b*, Insulin-Like Growth Factor 2*.* **b**. *igf1*, Insulin-Like Growth Factor 1. **c**. *gdf2.* Growth Differentiation Factor 2. **d**. *ctnnb1*, Catenin (Cadherin-Associated Protein), Beta 1. **e**. *gdf6a*, Growth Differentiation Factor 6. **f**. *igfbp2b*, Insulin-Like Growth Factor Binding Protein 2 (**g**). *igfbp5a*, Insulin-Like Growth Factor Binding Protein 5. **h**. *insra*, Insulin Receptor. **i**. *ppm1bb*, Protein Phosphatase, Mg2+/Mn2+ Dependent, 1B. **j**. *smad7*, SMAD Family Member 7

To validate the quality of RNA sequencing (RNA-Seq) data and the reliability of triploid expression level compared to both parents, we chose 10 representative differentially expressed genes (*igfbp2b*, *igfbp5a*, *smad7*, *gdf6a*, *igf1*, *ctnnb1*, *igf2b*, *ppm1bb*, *gdf2*, and *insra*) and performed qPCR on biological replicates in triplicate. The same trends in expression levels of these genes were detected by qPCR as were obtained from the RNA-Seq data analysis (Fig. 5). These results indicate that RNA-Seq data and associated analysis methods can be used to accurately detect differentially expressed genes.

## Discussion

### Dosage effect in triploid fish

To investigate whether a regulation mechanism was operating on gene dosage in a triploid genome context, 13,893 shared genes were used in our analysis because other genes may be errors in the assembly or the result of differential transcript expression in the nine individual livers. We compared the gene number of AT-ELV and PT-ELV among the total genes, and most of the total differentially expressed genes (4,048, 29.1% in total shared genes) were down-regulated; these results (number of up-regulated genes vs down-regulated genes = 1 vs 49.9), suggesting that dosage effects would occur in 4,048 genes of triploid (Fig. 3). A recent report suggested the silencing of one of three sets of alleles would result in transcript levels in triploid fish being decreased to the diploid state [10]. In contrast to both X chromosomes being partially repressed in *Caenorhabditis elegans* hermaphrodites (XX), the dosage effect resulted in the silencing of one X chromosome in vertebrates [34]. The existence of two identical sets of chromosomes in the nucleus would induce dosage compensation, which could result in the silencing of one set of maternal chromosomes (GC) in triploid (GB). The similar result was detected in salmon [17, 35]. However, the comparison of AT-ELV and PD-ELV was used to assess the percent of up- and down-regulated genes (see Methods). The results showed that the expression level (AT-ELV) of 2,232 genes was lower than ones’ in the diploid state (PD-ELV), while only 209 genes (AT-ELV) showed higher than ones’ in the diploid state (PD-ELV). That give insight into other mechanisms occurred in triploid offsprings, such as the level of methylation variation accompanied by triploidization, might act on the down-regulation of gene expression of some alleles, results in the expression level in triploid decreasing to lower than that in the diploid state [36]. The above results helped us understanding maternal GC-dosage effect on the parts of genes in triploid offspring.

### Homoeolog expression bias and expression level dominance

After charting the dosage effect in triploid fish, we used a novel method to analyze the state of expression levels between triploids and their diploid parents. This is the first report of this phenomenon in triploid fish (previously referred to as genomic dominance in plants [22, 32]). The 12 categories of expression level patterns were described above. Our results showed that the percentage of up-/down-regulated genes was 19.8% (2,749 genes) (Fig. 4a). Our method to analyze the negative dosage effect was feasible. Although we used the AT-ELV as the normalization state, the down- vs up-regulated ratio was 10.7:1 in triploid compared to the AT-ELV with PT-ELV (Fig. 4a). This suggested that the expression level of homoeologous genes was regulated after the genomes merged, which is the potential force behind the differential epigenetic regulation of the hybrid [22, 36]. Therefore, compared with the pattern of no change in initial predictions of triploid expression levels, the number of genes in the “no change” category was reduced because of the more feasible and detailed method that we used for classification based on expression levels.

To detect the global gene expression, the negative dosage effect of silencing of one set of maternal GC homoeologs was used in our analysis. Further analysis of triploid expression level compared with either of the diploid parents demonstrated the preferential transcription of maternal GC homoeologs in triploid (Fig. 4a). This phenomenon was commonly described in polyploidy [37] and refers to the pattern of redundant genes being silenced [38]. Approximately 25% of genes showed evidence of ELD in four allopolyploid cottons based on RNA-Seq data [22]. In triploid *Squalius alburnoides*, the *vasa* gene illustrated genome ELD in the gonad, and the *β-actin* gene exhibited the same phenomenon in the gonad and liver [10]. In addition to ELD, a second phenomenon was also described: middle expression levels were found in the polyploid based on the relative expression levels of the two parents. In our study, the 10,488 genes (75.5%, XI, XII, and No Change categories) showed expression levels that were regulated by homoeologs from both parents (Fig. 4a). These phenomena were always described in hybrids and polyploids based on the total gene analysis [22, 39, 40]. However, the phenomenon of middle expression levels exhibited organ-specific expression. For example, the *rpl8* and *gapdh* genes only show a mid-parents expression level in the liver of triploid individuals [10].

### Expression patterns of growth-related genes

The liver plays a major role in metabolism and has a number of functions, including the regulation of growth and development in fish. The study of the expression level of growth-related genes in triploid individuals is central to understanding the mechanism of the hybrid system. Here, we applied next-generation sequencing technology to study the relationship between growth rate and gene expression in a triploid. The dosage effect was evident in the global gene expression in the liver as we showed above. Genome-wide ELD shows maternal GC-HEB in the growth genes of triploid individuals.

In our study, 57 growth-related genes were screened from the categories of global gene expression (Fig. 4a). Four genes (7%) were up-regulated, which was higher than the percentage of total genes (0.5%) (*P* < 0.05; Fisher’s exact test). For example, *igfbp2b* and *igfbp5a* serve as a carrier protein for *igf-1*, which binds to *igf-1* inside the liver, allowing growth hormone to continuously act upon the liver to produce more *igf-1* [41]. Up-regulated expression of these genes will help organisms to accumulate and prolong the half-life of the insulin-like growth factors (Table 2; Fig. 5f and g). Another up-regulated gene, *igf-1*, and a paternal BSB-ELD gene, *igf2b*, were shown to play roles in the promotion of cell proliferation (Table 2; Fig. 5a and b). The up-regulated expression of *igf* in triploid was considered to play a crucial role in its faster growth rate relative to diploids [12]. The last up-regulated growth-related gene, *insra*, is a transmembrane receptor that is activated by insulin and *igf*, and it belongs to the class of tyrosine kinase receptors (Table 2; Fig. 5h) [42]. The up-regulation of *insra* resulted in an enhancement of the regulation of glucose homeostasis. Additionally, down-regulated expression of *ppm1bb* is known to be a negative regulator of cell stress response pathways, and overexpression of this phosphatase is reported to cause cell-growth arrest (Table 2; Fig. 5i) [43].

The other expression patterns were dominance and mid-parents expression level patterns that included 35 growth-related genes (Fig. 4a); these patterns provided insight into new expression level patterns in triploids. For example, paternal BSB-ELD was evident for the *gdf6* and *gdf2* genes (Table 2; Fig. 5c and e), which are members of the BMP family and the TGF-β superfamily that regulator cell growth and differentiation in both embryonic and adult tissues. These genes also promote bone and joint formation [44]. The hybrid individual had higher expression levels than maternal GC. These small changes in expression level contributed to changes in growth regulation. In addition, compared to the diploid GC, triploid had a significantly higher growth rate [6]. Therefore, these mechanisms might play important roles in the regulation of growth by changing some growth-related gene expression levels. Maternal GC-ELD gene *smad7* enhances muscle differentiation and plays a role in the negative feedback of TGF-β signaling (Table 2; Fig. 5j) [45]. These observations agreed with observations from some previous reports of polyploid fish [46, 47]. The middle-parents gene *ctnnb1* indicated that its expression was positively regulated by paternal BSB and resulted in up-regulated expression in triploid.

The differences in expression levels in triploid and the inbred diploid parents gave us a platform to investigate the rapid growth in triploid individuals. However, we should also investigate the gene expression changes that indirectly result in a change in growth traits. More research on these subjects will help us understand how the growth-related function was regulated in triploids. However, the observed results suggested that the rapid growth in triploids could be regulated by genes with a negative dosage effect.

### Mechanism of various expression patterns

Recent evidence showed that dosage compensation resulted in novel epigenetic regulation in triploids [17]. The current challenge is determining which changes in regulatory mechanisms explain the observed differences in gene expression levels and the evolution of complex phenotypes [35, 48]. Epigenetic instability in polyploids was described recently [49, 50]. Increased gene copy numbers from different species usually lead to changes in gene expression. This change usually destroys the steady state of the regulatory adaptations that were selected in the parents [50]. However, these abundant expression level patterns in polyploids provide important materials for adapting to various situations. The hybrids are likely to display regulatory alterations. These changes involved the silencing or activation of genes and DNA transposition of the Spm/CACTA family; these changes were described in allopolyploids of *Arabidopsis thaliana* [51, 52]. Our study also showed the activation of two genes in the liver transcriptome (Additional file 6). Possible mechanisms include small inhibitory RNA and epigenetic pathways that mediate the expression levels together with dosage compensation in triploids [35, 53].

## Conclusions

The hypothesis that differences in expression levels have an important role in speciation and adaptation has been accepted generally [48]. The mechanism of dosage compensation may be an extremely relevant factor contributing to the success and perpetuation of polyploidy in lower vertebrates [10]. Our results reveal the dosage effect occurring in triploid fish. To further analyze the regulated expression from dosage compensation, we used 12 expression patterns including up-/down-regulation, homoeolog dominance, and mid-parents to help us understand the speciation of triploid fish. The slightly unregulated growth genes and preferential transcription of paternal homoeologs provided insight into the regulation mechanisms that may contribute to the relationship between heterosis and growth expression in triploid fish. At present, we are trying to elaborate how these transcriptomic dynamics affect function and mediate phenotypes. In addition, the genes with changes in expression levels that were conferred by gene abundance are available for evolutionary experimentation. However, more studies using various species, tissues, and environmental conditions are needed to describe the various expression level patterns in hybrids and polyploids.

## Methods

### Animal material

For this study, all experiments were approved by Animal Care Committee of Hunan Normal University and followed guidelines statement of the Administration of Affairs Concerning Animal Experimentation of China. Experimental individuals were fed in a pool with suitable illumination, water temperature, dissolved oxygen content, and adequate forage for 19 months in the Engineering Center of Polyploidy Fish Breeding of the National Education Ministry located at Hunan Normal University, China. Triploid hybrids of female grass carp (*Ctenopharyngodon idellus*, GC, Cyprininae, 2n = 48) × male blunt snout bream (*Megalobrama amblycephala*, BSB, Cultrinae, 2n = 48) were successfully obtained by distant hybridization as a result of human selection (Fig. 1b and c) [6]. The 5S rDNA locus has been used to identify triploid hybrids that possessed 72 chromosomes with two sets from maternal GC and one set from paternal BSB [6]. Triploid hybrid of GC (♀) × BSB (♂) was abbreviated as GB hybrids. Nine individuals (three hybrids and six parents) were collected for our studies. The information about fish samples including body traits (body length, body height, and weight) and DNA content were obtained at the time of the experiment (Additional file 8).

The ploidy levels of the nine individuals were distinguished by a metaphase chromosome assay of cultured blood cells (Fig. 1a). After anesthetizing the fish with 2-phenoxyethanol, liver tissue was excised carefully to avoid gut contamination. The fish were treated humanely. All of the experiments were approved by the Animal Care Committee of Hunan Normal University and the Administration of Affairs Concerning Animal Experimentation guidelines stated approval from the Science and Technology Bureau of China. Samples were cut into small pieces and immediately pulled into RNALater (Ambion, AM7021, USA) at −80 °C following the manufacturer’s instructions. Total RNA was extracted from liver tissue of the BSB, GC, and GB samples. After RNALater was removed, the samples were homogenized using a pestle and mortar. RNA was isolated according to the standard trizol protocol, and agarose gel electrophoresis and the optical density at 260 nm (OD260)/OD280 ratio was used to assess RNA quality. A TURBO DNA-free kit was used to remove DNA contamination.

### Illumina sequencing and assembly of the Illumina contigs

Poly (A) mRNA isolation was performed using oligo (dT) beads after total RNA collection. Fragmentation buffer was added to generate short fragments of mRNA. Using these short fragments as templates, first-strand cDNA was synthesized by a random hexamer primer. Second-strand cDNA was then synthesized using buffer, dNTPs, RNaseH, and DNA polymerase I. Short fragments were purified with the QiaQuick PCR extraction kit (Qiagen) and resolved with elution buffer. These fragments were separated by agarose gel electrophoresis after adding sequencing adapters. PCR amplification templates of the suitable fragments were selected. During the quality control steps, the Agilent 2100 Bioanalyzer and ABI StepOnePlus Real-Time PCR System were used to qualify and quantify the sample library. Finally, the nine libraries from the nine individuals (six parents and three triploids) were sequenced using an Illumina HiSeq™ 2000/2500.

After raw reads were produced by sequencing, the read adaptors and low quality reads were removed. Transcriptome *de novo* assembly was carried out with a short-reads assembly program (Trinity) [54], using three independent software modules called Inchworm, Chrysalis, and Butterfly. Principal component analysis (PCA) of nine liver transcriptomes was applied to examine the contribution of each transcript to the separation of the classes [55, 56] (Additional file 9).

### Gene annotation

Contig annotation was performed using the five public databases. BLASTX alignment (e-value ≤ 1e^−6^) between contigs and protein databases was performed, and the best-aligned results were used to decide the sequence direction of contigs (Additional file 1). After screening the sequences (alignment length ≤ 100 bp), accession numbers of the genes were obtained from the BLASTX results. Then, GO terms of annotation sequences were obtained through Ensembl BioMart [57]. WEGO software was used to analyze the GO annotation (Additional file 2) [58]. For pathway enrichment analysis, we mapped all differentially expressed genes to terms in the KEGG database and looked for significantly enriched KEGG terms (Additional file 10).

### Mapping and differential expression

To obtain the shared transcripts in the three species, the reference transcripts were merged from the BSB, GC, and GB contigs using CD-HIT with 95% as the threshold [59]. Then, we utilized the merged sequences as the reference transcript because this database was built using transcripts from both parents and the hybrid offspring. The total clean reads were aligned against the merged sequences using Blat [33]. Then, information about the expression level in the three species was reflected by the number of aligned reads.

Mapped, filtered, and sorted reads were analyzed with the DEGseq package in R software version 2.13 (R Foundation for Statistical Computing, Vienna, Austria) [60]. Differential expression was assessed in triploids and their diploid parents using Fisher’s exact tests [61]. The abundance or the coverage of each transcript was determined by read counts and normalized using the number of reads per kilobase exon per million mapped reads (RPKM) [62]. The RPKM value of the read density reflected the molar concentration of a transcript in the starting sample after normalizing for the RNA length and total read number in the measurements. This facilitated a transparent comparison of transcript levels within and between samples. Herein, we defined gene expression as the average sequence expression of a gene, and a species comparison was shown (Fig. 3a, b, and c).

### Dosage compensation in triploid fish

The absolute values of the log_2_Ratio ≤ 1 were used as the threshold to judge the significance of the gene expression difference. Expression values above the threshold were described as upregulated and those below the threshold were described as downregulated.

To effectively analyze the dosage effects in triploid, we first set the PT-ELV (χ_triploid_) according to the composition of the genome: two sets of genomes from maternal GC and one set from paternal BSB [6]. The value was constructed from two parts in which one is half the BSB value of gene expression (χ_BSB_) and the other is the GC values of gene expression (χ_GC_) (χ_triploid_ = 1/2χ_BSB_ + χ_GC_). If no dosage effect happens in triploids, the gene expression level of triploids will float along with χ_triploid_. However, comparing the AT-ELV with PT-ELV in triploids revealed that most genes were down-regulated. In this situation, we assumed that the dosage effect occurred in maternal GC homoeolog of triploids similar to other triploid individuals [10] and set up the PD-ELV (χ_diploid_ = 1/2χ_BSB_ + 1/2χ_GC_). Comparing the AT-ELV and PD-ELV, the number of differentially expressed genes showed trends of up- and down-regulation in triploid fish.

### Analyses of expression level dominance and homoeolog expression bias

We explored the data to identify candidate novel expression (new expression of a gene in liver) and homoeolog silencing patterns (no expression of one homoeolog) in the hybrids. Novel expression was inferred when both parental species had no reads for a gene, yet hybrids displayed more than 10 RPKM. If both parental species had more than 10 RPKM, but hybrids had zero counts for the same gene, this was considered silencing. These two cases were eliminated from further analysis, and we focused on genes that were expressed among both the diploid parents and triploid offspring.

In triploid offspring, the total liver genes were affected by a negative dosage effect. Genes that were identified as differentially expressed in the hybrid relative to the diploid parents were binned into 12 possible expression classes of differential expression (Fig. 4a), ELD, mid-parents, and up/down expression (outside the range of either parent), according to Rappet et al*.* (2009) [32]. Briefly, genes were parsed into these 12 categories (using Roman numerals; see Fig. 4a), depending on the relative expression levels between triploid and the diploid parents. Examined in this manner, genes may display mid-parents (XI and XII), paternal BSB-ELD (VII and VIII), maternal GC-ELD (IX and X), expression lower than both parents (I, II, and III), or expression higher than both parents (IV, V, and VI). For each of the 12 categories above (which are based on joint expression levels for both homoeologs), we calculated the RPKM value of reads to examine the gene expression for each homoeolog pair. The FDR was used to determine the threshold P value in multiple tests and analyses. FDR < 0.001 and the absolute value of log_2_ ratio ≤ 1 were used as thresholds to judge the significance of gene expression differences between two species. For each gene, the expression level of the two diploid parents was estimated and classified into three situations; then, the expression level of triploid hybrid for the same gene was exhibited in the three situations (Fig. 4b, c, and d).

### qPCR analysis

According to the expression level of transcriptome data, we had detected the expression of *β-actin* among of BSB, GC and GB. The expression level of *β-actin* in liver of triploid was also decreased to one’s in diploid state. So *β-actin* could be considered as the references gene in qPCR. The total RNA that was extracted from the liver tissue was used for qPCR analysis. qPCR analysis was performed using the Prism 7500 Sequence Detection System (Applied Biosystems) with a miScript SYBR Green PCR kit (Qiagen). qPCR was performed on biological replicates in triplicate (and triplicate technical qPCR replicates). The amplification conditions were as follows: 50 °C for 5 min and 95 °C for 10 min, followed by 40 cycles at 95 °C for 15 s and 60 °C for 45 s. The average threshold cycle (Ct) was calculated for each sample using the 2^-ΔΔCt^ method and normalized to *β-actin*. Lastly, a melting curve analysis was completed to validate the specific generation of the expected product.

## Additional files

Additional file 1: Summary information of assembled sequences blasted against the five databases. (A). Contig distribution of GC, BSB and GB, and merge sequences aligned to NCBI-NR, Swiss-Prot, KEGG, COG and GO, respectively. (B). E-value distribution of BLASTX hits with threshold of 1.0E-6. (TIF 647 kb)
Additional file 2: Gene ontology (GO) assignments for the GC, BSB and GB. GO assignments (level 2) were used to predict the functional distribution including cellular component ontology, molecular function ontology and biological processes ontology. (TIF 3893 kb)
Additional file 3: Venn diagram where the area of each circle (and intersections) is proportional to the number of unigenes from GC, BSB and GB after GO annotation. Numbers are indicated in each section. (TIF 561 kb)
Additional file 4: Distribution of contigs of GC, BSB and GB and merge sequences. The 95,702 contigs were merged using CD-HIT. (TIF 215 kb)
Additional file 5: The basic information of mapping data. (DOCX 17 kb)
Additional file 6: The basic information of gene silencing and novel genes in triploid offspring. (DOCX 19 kb)
Additional file 7: Basic information of the categories of “No change” in growth genes as comparison of the triploid hybrids with its parents. (DOCX 17 kb)
Additional file 8: Comparison of the measurable traits among the hybrid offspring and their parents. (DOCX 20 kb)
Additional file 9: (Color online) Symmetric heatmap of attribute correlations among nine individuals. Blue (red) indicates perfect correlation (anti-correlation). White exhibits the intermediate case of no correlation. The small amount of clustering along the diagonal attests to the relative independence of the attributes. (TIF 182 kb)
Additional file 10: The pathway information in three species. (DOCX 30 kb)

## Abbreviations

Blat: BLAST-like alignment tool
AT-ELV: The value of actual triploid expression level
ELD: Expression level dominances
FDR: False discovery rate
HEB: Homoeolog expression bias
PCA: Principal component analysis
PD-ELV: The value of predicted diploid expression level
PT-ELV: The value of predicted triploid expression level
RPKM: Reads per kilobase exon per million mapped reads
RT-qPCR: Real-time quantitative polymerase chain reaction
