# Supplementary figures and images for: Determination of dosage compensation and comparison of gene expression in a triploid hybrid fish
Source: BMC Genomics. 2017 Jan 5;18:38. doi: 10.1186/s12864-016-3424-5 (PMC5216571; doi:10.1186/s12864-016-3424-5)

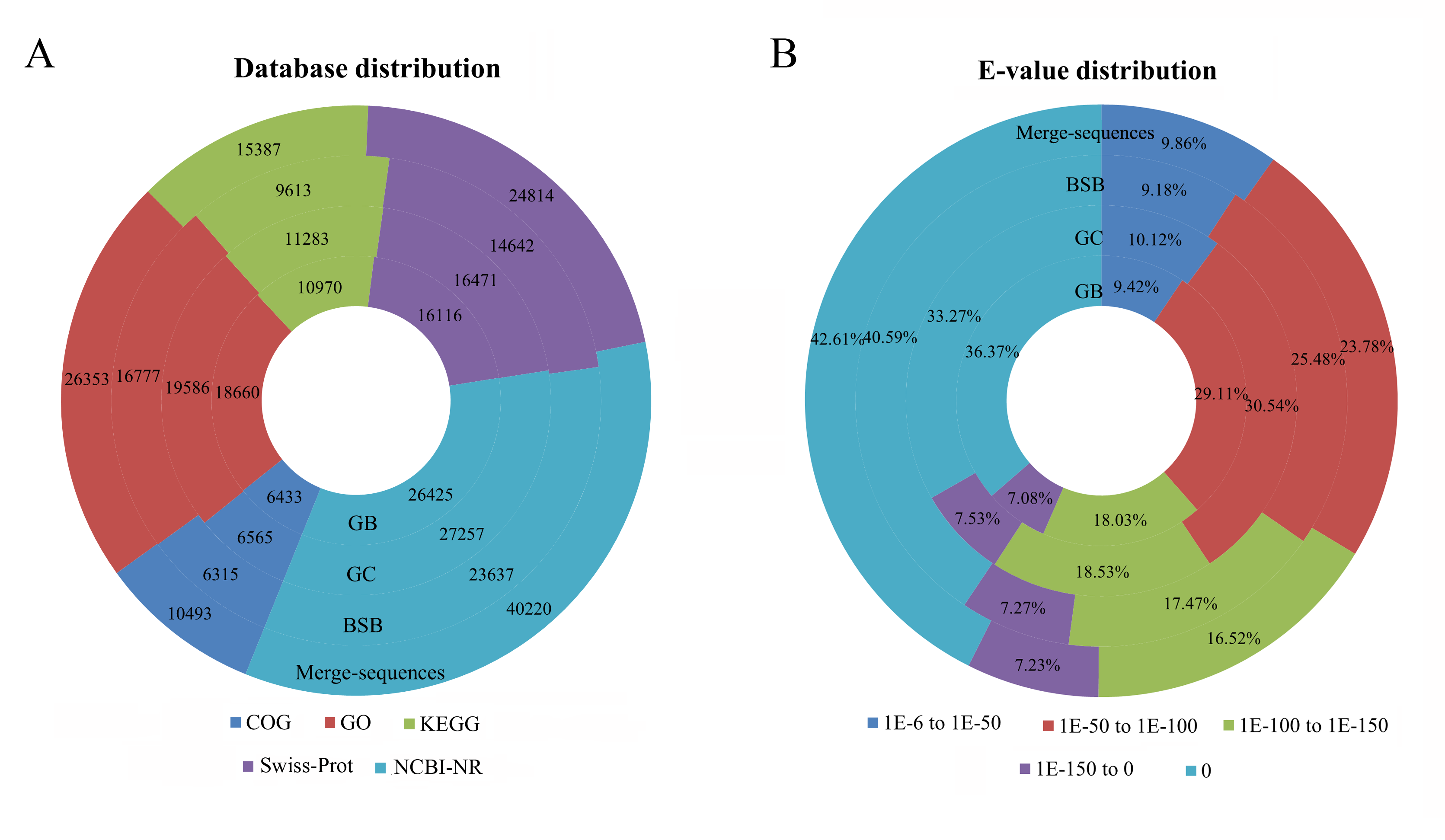

Supplement: Additional file 1: — Summary information of assembled sequences blasted against the five databases. (A). Contig distribution of GC, BSB and GB, and merge sequences aligned to NCBI-NR, Swiss-Prot, KEGG, COG and GO, respectively. (B). E-value distribution of BLASTX hits with threshold of 1.0E-6. (TIF 647 kb) [file 12864_2016_3424_MOESM1_ESM.tif]

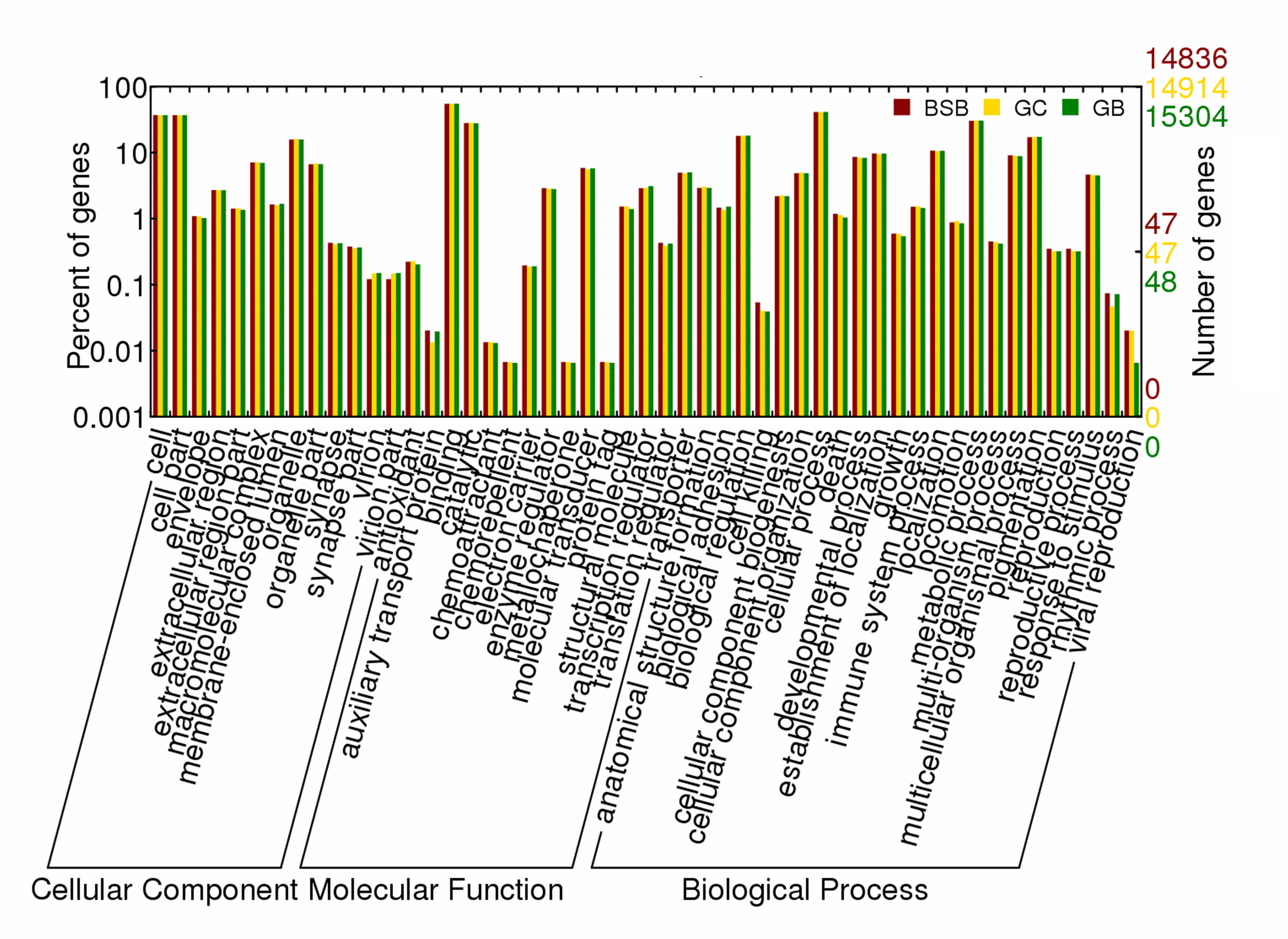

Supplement: Additional file 2: — Gene ontology (GO) assignments for the GC, BSB and GB. GO assignments (level 2) were used to predict the functional distribution including cellular component ontology, molecular function ontology and biological processes ontology. (TIF 3893 kb) [file 12864_2016_3424_MOESM2_ESM.tif]

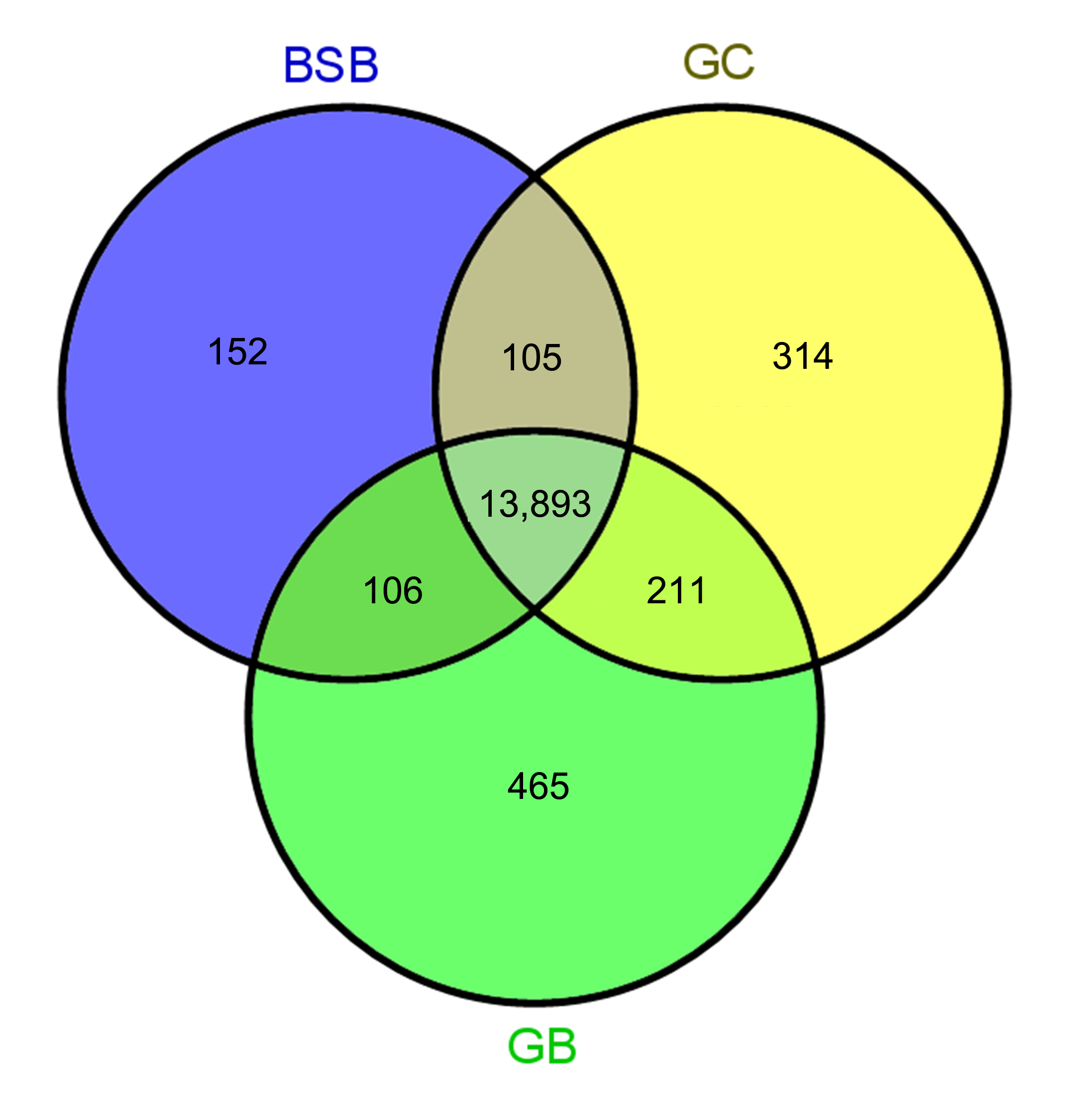

Supplement: Additional file 3: — Venn diagram where the area of each circle (and intersections) is proportional to the number of unigenes from GC, BSB and GB after GO annotation. Numbers are indicated in each section. (TIF 561 kb) [file 12864_2016_3424_MOESM3_ESM.tif]

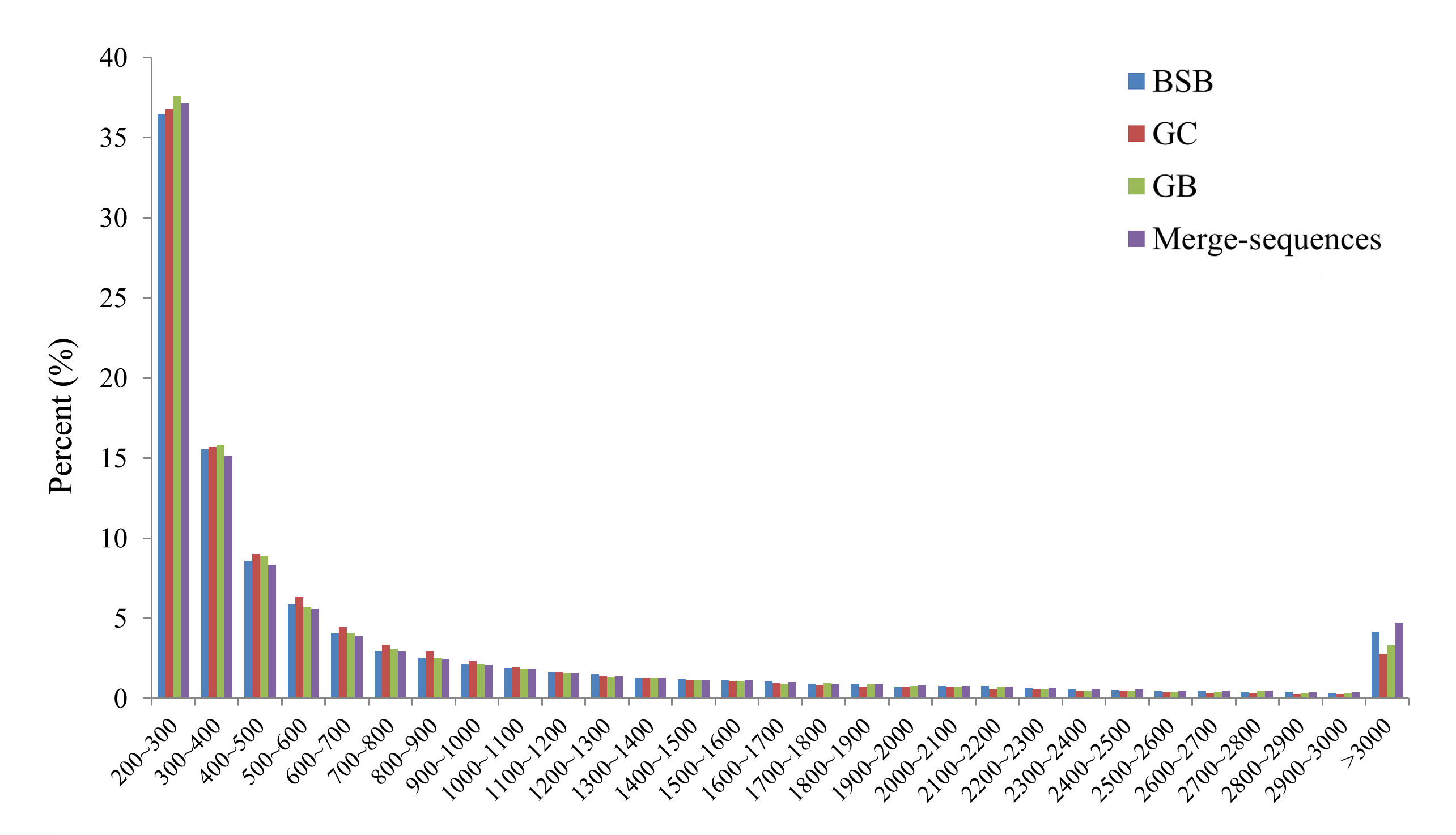

Supplement: Additional file 4: — Distribution of contigs of GC, BSB and GB and merge sequences. The 95,702 contigs were merged using CD-HIT. (TIF 215 kb) [file 12864_2016_3424_MOESM4_ESM.tif]

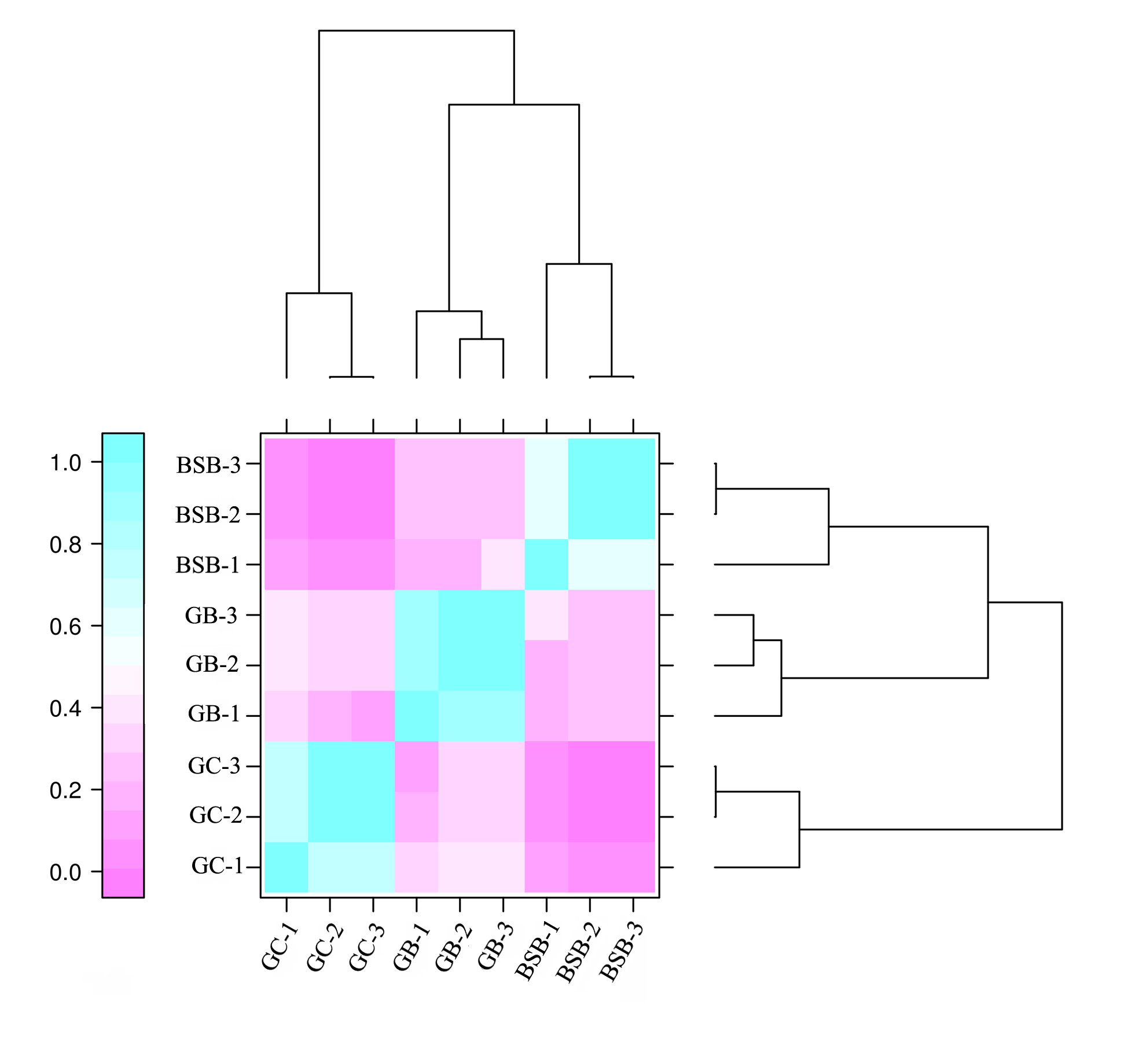

Supplement: Additional file 9: — (Color online) Symmetric heatmap of attribute correlations among nine individuals. Blue (red) indicates perfect correlation (anti-correlation). White exhibits the intermediate case of no correlation. The small amount of clustering along the diagonal attests to the relative independence of the attributes. (TIF 182 kb) [file 12864_2016_3424_MOESM9_ESM.tif]
