# Supplementary material for: Determination of dosage compensation and comparison of gene expression in a triploid hybrid fish
Source: BMC Genomics. 2017 Jan 5;18:38. doi: 10.1186/s12864-016-3424-5 (PMC5216571; doi:10.1186/s12864-016-3424-5)
Supplement: Additional file 5: — The basic information of mapping data. (DOCX 17 kb) [file 12864_2016_3424_MOESM5_ESM.docx]

**Table S1. The basic information of mapping data**

| Sample | Total reads | Total mapping reads | Uniquely mapping reads | Multiply mapping reads |
| --- | --- | --- | --- | --- |
| BSB-1 | 27,643,051 | 23,570,060 (85.3%) | 14,105,556 (59.8%) | 9,464,504 (40.2%) |
| BSB-2 | 23,693,226 | 18,697,304 (78.9%) | 10,816,254 (57.8%) | 7,881,050 (42.2%) |
| BSB-3 | 21,164,604 | 16,713,029 (79.0%) | 9,651,158 (57.7%) | 7,061,871 (42.3%) |
| GC-1 | 25,801,874 | 21,120,138 (81.9%) | 13,669,055 (64.7%) | 7,451,083 (35.3%) |
| GC-2 | 23,518,445 | 19,643,068 (83.5%) | 11,860,575 (60.4%) | 7,782,493 (39.6%) |
| GC-3 | 21,650,846 | 17,829,888 (82.4%) | 10,840,811 (60.8%) | 6,989,077 (39.2%) |
| GB-1 | 27,666,666 | 23,324,536 (84.3%) | 14,787,542 (63.4%) | 8,536,994 (36.6%) |
| GB-2 | 23,625,506 | 18,685,584 (79.1%) | 11,207,918 (50.0%) | 7,477,666 (40.0%) |
| GB-3 | 23,156,195 | 18,486,797 (79.8%) | 11,023,979 (59.6%) | 7,462,818 (40.4%) |
