# Supplementary material for: Determination of dosage compensation and comparison of gene expression in a triploid hybrid fish
Source: BMC Genomics. 2017 Jan 5;18:38. doi: 10.1186/s12864-016-3424-5 (PMC5216571; doi:10.1186/s12864-016-3424-5)
Supplement: Additional file 6: — The basic information of gene silencing and novel genes in triploid offspring. (DOCX 19 kb) [file 12864_2016_3424_MOESM6_ESM.docx]

**Table S2. The basic information of gene silencing and novel genes in triploid offspring.**

| Gene type | Gene symbol | GB count | BSB count | GC count |
| --- | --- | --- | --- | --- |
| Silencing genes | *si:dkey-19a16.1* | 0 | 331 | 1214 |
|  | *cu596012.1* | 0 | 130 | 0 |
|  | *bfb* | 0 | 196 | 0 |
|  | *ccna1* | 0 | 0 | 102 |
|  | *sycp1* | 0 | 0 | 102 |
|  | *ccdc19* | 0 | 0 | 105 |
|  | *ccdc39* | 0 | 0 | 107 |
|  | *bx664721.1* | 0 | 0 | 107 |
|  | *ropn1l* | 0 | 0 | 110 |
|  | *si:ch211-199g17.9* | 0 | 0 | 111 |
|  | *ccdc42b* | 0 | 0 | 112 |
|  | *c5h9orf9* | 0 | 0 | 122 |
|  | *ccdc173* | 0 | 0 | 124 |
|  | *dmc1* | 0 | 0 | 124 |
|  | *dynlrb2* | 0 | 0 | 136 |
|  | *ribc1* | 0 | 0 | 139 |
|  | *prf1.5* | 0 | 0 | 160 |
|  | *sept4a* | 0 | 0 | 168 |
|  | *si:ch211-71m22.1* | 0 | 0 | 179 |
|  | *rsph9* | 0 | 0 | 181 |
|  | *stmn1a* | 0 | 0 | 198 |
|  | *rsph1* | 0 | 0 | 204 |
|  | *ccdc114* | 0 | 0 | 266 |
|  | *odf3b* | 0 | 0 | 384 |
|  | *neil3* | 0 | 0 | 414 |
|  | *tubb4b* | 0 | 0 | 415 |
|  | *hmgb2b* | 0 | 0 | 500 |
| Novel genes | *ca15b* | 388 | 0 | 0 |
|  | *cu694951.3* | 427 | 0 | 0 |
