# Supplementary material for: Determination of dosage compensation and comparison of gene expression in a triploid hybrid fish
Source: BMC Genomics. 2017 Jan 5;18:38. doi: 10.1186/s12864-016-3424-5 (PMC5216571; doi:10.1186/s12864-016-3424-5)
Supplement: Additional file 7: — Basic information of the categories of “No change” in growth genes as comparison of the triploid hybrids with its parents. (DOCX 17 kb) [file 12864_2016_3424_MOESM7_ESM.docx]

**Table S5. Basic information of the categories of “No change” in growth genes as comparison of the triploid hybrids with its parents**

| Categories | Ensembl protein ID | BSB-RPKM | GB-RPKM | GC-RPKM | Symbols | Go term name | Go term accession |
| --- | --- | --- | --- | --- | --- | --- | --- |
| No change | ENSDARP00000043454 | 4.31 | 2.18 | 2.06 | *smad3b* | transforming growth factor beta receptor signaling pathway | GO:0007179 |
| No change | ENSDARP00000037789 | 7.11 | 7.00 | 5.51 | *klf6a* | organ growth | GO:0035265 |
| No change | ENSDARP00000009432 | 9.04 | 8.92 | 14.70 | *cnpy1* | fibroblast growth factor receptor signaling pathway, regulation of fibroblast growth factor receptor signaling pathway | GO:0008543 GO:0040036 |
| No change | ENSDARP00000047861 | 0.84 | 0.50 | 0.35 | *si:dkey-222b8.1* | transforming growth factor beta receptor signaling pathway | GO:0007179 |
| No change | ENSDARP00000103396 | 37.04 | 32.56 | 44.67 | *rabep2* | growth factor activity | GO:0008083 |
| No change | ENSDARP00000016954 | 16.76 | 9.73 | 24.56 | *igf2a* | growth factor activity, insulin-like growth factor receptor binding | GO:0008083 GO:0005159 |
| No change | ENSDARP00000109240 | 4.30 | 3.25 | 6.69 | *bx511266.1* | growth factor activity | GO:0008083 |
| No change | ENSDARP00000105258 | 4.27 | 3.77 | 4.27 | *smad5* | transforming growth factor beta receptor signaling pathway | GO:0007179 |
| No change | ENSDARP00000018598 | 3.27 | 1.75 | 1.11 | *mul1a* | negative regulation of cell growth | GO:0030308 |
| No change | ENSDARP00000105972 | 2.19 | 2.79 | 4.10 | *igfbp5b* | regulation of cell growth, insulin-like growth factor binding | GO:0001558 GO:0005520 |
| No change | ENSDARP00000068816 | 13.80 | 11.05 | 16.93 | *igfbp2a* | regulation of cell growth, growth, regulation of growth, regulation of insulin-like growth factor receptor signaling pathway, negative regulation of developmental growth, negative regulation of multicellular organism growth, insulin-like growth factor binding, growth factor binding, insulin-like growth factor I binding, insulin-like growth factor II binding | GO:0001558 GO:0040007 GO:0040008 GO:0043567 GO:0048640 GO:0040015 GO:0005520 GO:0019838 GO:0031994 GO:0031995 |
| No change | ENSDARP00000019674 | 3.11 | 4.81 | 6.35 | *igfbp1a* | regulation of cell growth, insulin-like growth factor binding, insulin-like growth factor I binding, insulin-like growth factor II binding | GO:0001558 GO:0005520 GO:0031994 GO:0031995 |
