# Supplementary material for: Determination of dosage compensation and comparison of gene expression in a triploid hybrid fish
Source: BMC Genomics. 2017 Jan 5;18:38. doi: 10.1186/s12864-016-3424-5 (PMC5216571; doi:10.1186/s12864-016-3424-5)
Supplement: Additional file 8: — Comparison of the measurable traits among the hybrid offspring and their parents. (DOCX 20 kb) [file 12864_2016_3424_MOESM8_ESM.docx]

**Table S3. Comparison of the measurable traits among the hybrid offspring and their parents**

| Fish type | Age (month) | Sex | Weight (kg) | BL/WL | BW/BL | HL/BL | HW/HL | TW/TL | HW/BW |
| --- | --- | --- | --- | --- | --- | --- | --- | --- | --- |
| GC | 19 | [female](javascript:void(0);) | 2.7± 0.54 | 0.84 ± 0.04 | 0.26 ± 0.04 | 0.25 ± 0.04 | 0.77 ± 0.03 | 0.86 ± 0.03 | 0.73 ± 0.03 |
| BSB | 19 | [female](javascript:void(0);) | 2.4± 0.34 | 0.84 ± 0.03 | 0.42 ± 0.03 | 0.21 ± 0.04 | 0.88 ± 0.03 | 0.93 ± 0.04 | 0.48 ± 0.04 |
| GB | 19 | [female](javascript:void(0);) | 4.6± 0.84 | 0.83 ± 0.02 | 0.27 ± 0.01 | 0.22 ± 0.02 | 0.81 ± 0.08 | 0.93 ± 0.07 | 0.67 ± 0.06 |
| P value (GC vs GB) |  |  | < 0.05 | > 0.05 | < 0.01 | > 0.05 | > 0.05 | > 0.05 | < 0.01 |
| P value (BSB vs GB) |  |  | < 0.01 | > 0.05 | > 0.05 | > 0.05 | > 0.05 | > 0.05 | < 0.05 |

The information of measurable traits including the average values of whole length (WL), body length (BL), body width (BW), head length (HL), head width (HW), tail length (TL) and tail width (TW) were obtained. In addition, the average ratios of body length to whole length (BL/WL), body width to body length (BW/BL), head length to body length (HL/BL), head width to head length (HW/HL), tail width to tail length (TW/TL) and head width to body width (HW/BW) were also calculated.
