# Supplementary material for: Determination of dosage compensation and comparison of gene expression in a triploid hybrid fish
Source: BMC Genomics. 2017 Jan 5;18:38. doi: 10.1186/s12864-016-3424-5 (PMC5216571; doi:10.1186/s12864-016-3424-5)
Supplement: Additional file 10: — The pathway information in three species. (DOCX 30 kb) [file 12864_2016_3424_MOESM10_ESM.docx]

**Table S4. The pathway information in three species**

| #Pathway name | Pathway ID | Gene number |
| --- | --- | --- |
| Glycolysis / Gluconeogenesis | ko00010 | 123 |
| Citrate cycle (TCA cycle) | ko00020 | 71 |
| Pentose phosphate pathway | ko00030 | 56 |
| Pentose and glucuronate interconversions | ko00040 | 36 |
| Fructose and mannose metabolism | ko00051 | 87 |
| Galactose metabolism | ko00052 | 48 |
| Ascorbate and aldarate metabolism | ko00053 | 25 |
| Fatty acid biosynthesis | ko00061 | 14 |
| Fatty acid elongation in mitochondria | ko00062 | 18 |
| Fatty acid metabolism | ko00071 | 95 |
| Synthesis and degradation of ketone bodies | ko00072 | 14 |
| Steroid biosynthesis | ko00100 | 33 |
| Primary bile acid biosynthesis | ko00120 | 22 |
| Ubiquinone and other terpenoid-quinone biosynthesis | ko00130 | 12 |
| Steroid hormone biosynthesis | ko00140 | 48 |
| Oxidative phosphorylation | ko00190 | 270 |
| Purine metabolism | ko00230 | 350 |
| Caffeine metabolism | ko00232 | 5 |
| Pyrimidine metabolism | ko00240 | 203 |
| Alanine, aspartate and glutamate metabolism | ko00250 | 65 |
| Glycine, serine and threonine metabolism | ko00260 | 63 |
| Cysteine and methionine metabolism | ko00270 | 75 |
| Valine, leucine and isoleucine degradation | ko00280 | 91 |
| Geraniol degradation | ko00281 | 1 |
| Valine, leucine and isoleucine biosynthesis | ko00290 | 18 |
| Lysine biosynthesis | ko00300 | 7 |
| Lysine degradation | ko00310 | 127 |
| Arginine and proline metabolism | ko00330 | 106 |
| Histidine metabolism | ko00340 | 44 |
| Tyrosine metabolism | ko00350 | 43 |
| Phenylalanine metabolism | ko00360 | 26 |
| Benzoate degradation | ko00362 | 1 |
| Tryptophan metabolism | ko00380 | 75 |
| Phenylalanine, tyrosine and tryptophan biosynthesis | ko00400 | 13 |
| beta-Alanine metabolism | ko00410 | 48 |
| Taurine and hypotaurine metabolism | ko00430 | 13 |
| Selenocompound metabolism | ko00450 | 35 |
| Cyanoamino acid metabolism | ko00460 | 15 |
| D-Glutamine and D-glutamate metabolism | ko00471 | 12 |
| D-Arginine and D-ornithine metabolism | ko00472 | 3 |
| Glutathione metabolism | ko00480 | 73 |
| Starch and sucrose metabolism | ko00500 | 73 |
| N-Glycan biosynthesis | ko00510 | 93 |
| Other glycan degradation | ko00511 | 34 |
| Mucin type O-Glycan biosynthesis | ko00512 | 35 |
| Other types of O-glycan biosynthesis | ko00514 | 51 |
| Amino sugar and nucleotide sugar metabolism | ko00520 | 86 |
| Butirosin and neomycin biosynthesis | ko00524 | 5 |
| Glycosaminoglycan degradation | ko00531 | 32 |
| Glycosaminoglycan biosynthesis - chondroitin sulfate | ko00532 | 37 |
| Glycosaminoglycan biosynthesis - keratan sulfate | ko00533 | 29 |
| Glycosaminoglycan biosynthesis - heparan sulfate | ko00534 | 47 |
| Glycerolipid metabolism | ko00561 | 105 |
| Inositol phosphate metabolism | ko00562 | 145 |
| Glycosylphosphatidylinositol(GPI)-anchor biosynthesis | ko00563 | 49 |
| Glycerophospholipid metabolism | ko00564 | 179 |
| Ether lipid metabolism | ko00565 | 56 |
| Arachidonic acid metabolism | ko00590 | 67 |
| Linoleic acid metabolism | ko00591 | 27 |
| alpha-Linolenic acid metabolism | ko00592 | 18 |
| Sphingolipid metabolism | ko00600 | 80 |
| Glycosphingolipid biosynthesis - lacto and neolacto series | ko00601 | 34 |
| Glycosphingolipid biosynthesis - globo series | ko00603 | 9 |
| Glycosphingolipid biosynthesis - ganglio series | ko00604 | 18 |
| Pyruvate metabolism | ko00620 | 85 |
| Glyoxylate and dicarboxylate metabolism | ko00630 | 34 |
| Propanoate metabolism | ko00640 | 73 |
| Ethylbenzene degradation | ko00642 | 1 |
| Butanoate metabolism | ko00650 | 43 |
| One carbon pool by folate | ko00670 | 35 |
| Thiamine metabolism | ko00730 | 6 |
| Riboflavin metabolism | ko00740 | 22 |
| Vitamin B6 metabolism | ko00750 | 10 |
| Nicotinate and nicotinamide metabolism | ko00760 | 47 |
| Pantothenate and CoA biosynthesis | ko00770 | 32 |
| Biotin metabolism | ko00780 | 5 |
| Lipoic acid metabolism | ko00785 | 4 |
| Folate biosynthesis | ko00790 | 18 |
| Retinol metabolism | ko00830 | 42 |
| Porphyrin and chlorophyll metabolism | ko00860 | 63 |
| Terpenoid backbone biosynthesis | ko00900 | 24 |
| Nitrogen metabolism | ko00910 | 37 |
| Sulfur metabolism | ko00920 | 12 |
| Aminoacyl-tRNA biosynthesis | ko00970 | 64 |
| Metabolism of xenobiotics by cytochrome P450 | ko00980 | 43 |
| Drug metabolism - cytochrome P450 | ko00982 | 40 |
| Drug metabolism - other enzymes | ko00983 | 55 |
| Biosynthesis of unsaturated fatty acids | ko01040 | 31 |
| ABC transporters | ko02010 | 91 |
| Two-component system | ko02020 | 4 |
| Phosphotransferase system (PTS) | ko02060 | 1 |
| Ribosome biogenesis in eukaryotes | ko03008 | 148 |
| Ribosome | ko03010 | 234 |
| RNA transport | ko03013 | 307 |
| mRNA surveillance pathway | ko03015 | 162 |
| RNA degradation | ko03018 | 148 |
| RNA polymerase | ko03020 | 56 |
| Basal transcription factors | ko03022 | 64 |
| DNA replication | ko03030 | 103 |
| Spliceosome | ko03040 | 268 |
| Proteasome | ko03050 | 84 |
| Protein export | ko03060 | 35 |
| Bacterial secretion system | ko03070 | 1 |
| PPAR signaling pathway | ko03320 | 129 |
| Base excision repair | ko03410 | 77 |
| Nucleotide excision repair | ko03420 | 103 |
| Mismatch repair | ko03430 | 71 |
| Homologous recombination | ko03440 | 62 |
| Non-homologous end-joining | ko03450 | 45 |
| MAPK signaling pathway | ko04010 | 507 |
| MAPK signaling pathway - yeast | ko04011 | 1 |
| ErbB signaling pathway | ko04012 | 203 |
| MAPK signaling pathway - fly | ko04013 | 2 |
| Calcium signaling pathway | ko04020 | 312 |
| Cytokine-cytokine receptor interaction | ko04060 | 272 |
| Chemokine signaling pathway | ko04062 | 39 |
| Phosphatidylinositol signaling system | ko04070 | 232 |
| Neuroactive ligand-receptor interaction | ko04080 | 215 |
| Cell cycle | ko04110 | 339 |
| Oocyte meiosis | ko04114 | 312 |
| p53 signaling pathway | ko04115 | 146 |
| Ubiquitin mediated proteolysis | ko04120 | 279 |
| Sulfur relay system | ko04122 | 13 |
| SNARE interactions in vesicular transport | ko04130 | 59 |
| Regulation of autophagy | ko04140 | 37 |
| Protein processing in endoplasmic reticulum | ko04141 | 311 |
| Lysosome | ko04142 | 234 |
| Endocytosis | ko04144 | 503 |
| Phagosome | ko04145 | 250 |
| Peroxisome | ko04146 | 156 |
| mTOR signaling pathway | ko04150 | 124 |
| Apoptosis | ko04210 | 189 |
| Cardiac muscle contraction | ko04260 | 155 |
| Vascular smooth muscle contraction | ko04270 | 263 |
| Wnt signaling pathway | ko04310 | 368 |
| Dorso-ventral axis formation | ko04320 | 58 |
| Notch signaling pathway | ko04330 | 124 |
| Hedgehog signaling pathway | ko04340 | 89 |
| TGF-beta signaling pathway | ko04350 | 167 |
| Axon guidance | ko04360 | 20 |
| VEGF signaling pathway | ko04370 | 171 |
| Osteoclast differentiation | ko04380 | 15 |
| Focal adhesion | ko04510 | 503 |
| ECM-receptor interaction | ko04512 | 192 |
| Cell adhesion molecules (CAMs) | ko04514 | 210 |
| Adherens junction | ko04520 | 243 |
| Tight junction | ko04530 | 285 |
| Gap junction | ko04540 | 201 |
| Complement and coagulation cascades | ko04610 | 4 |
| Antigen processing and presentation | ko04612 | 10 |
| Renin-angiotensin system | ko04614 | 21 |
| Toll-like receptor signaling pathway | ko04620 | 165 |
| NOD-like receptor signaling pathway | ko04621 | 108 |
| RIG-I-like receptor signaling pathway | ko04622 | 113 |
| Cytosolic DNA-sensing pathway | ko04623 | 73 |
| Jak-STAT signaling pathway | ko04630 | 219 |
| Hematopoietic cell lineage | ko04640 | 10 |
| Natural killer cell mediated cytotoxicity | ko04650 | 188 |
| T cell receptor signaling pathway | ko04660 | 22 |
| B cell receptor signaling pathway | ko04662 | 16 |
| Fc epsilon RI signaling pathway | ko04664 | 9 |
| Fc gamma R-mediated phagocytosis | ko04666 | 23 |
| Leukocyte transendothelial migration | ko04670 | 20 |
| Intestinal immune network for IgA production | ko04672 | 47 |
| Circadian rhythm - mammal | ko04710 | 67 |
| Long-term potentiation | ko04720 | 30 |
| Neurotrophin signaling pathway | ko04722 | 23 |
| Long-term depression | ko04730 | 22 |
| Olfactory transduction | ko04740 | 9 |
| Taste transduction | ko04742 | 5 |
| Phototransduction | ko04744 | 14 |
| Regulation of actin cytoskeleton | ko04810 | 482 |
| Insulin signaling pathway | ko04910 | 322 |
| GnRH signaling pathway | ko04912 | 222 |
| Progesterone-mediated oocyte maturation | ko04914 | 233 |
| Melanogenesis | ko04916 | 241 |
| Adipocytokine signaling pathway | ko04920 | 166 |
| Type II diabetes mellitus | ko04930 | 3 |
| Type I diabetes mellitus | ko04940 | 3 |
| Aldosterone-regulated sodium reabsorption | ko04960 | 4 |
| Vasopressin-regulated water reabsorption | ko04962 | 3 |
| Proximal tubule bicarbonate reclamation | ko04964 | 2 |
| Salivary secretion | ko04970 | 20 |
| Gastric acid secretion | ko04971 | 25 |
| Pancreatic secretion | ko04972 | 16 |
| Carbohydrate digestion and absorption | ko04973 | 5 |
| Protein digestion and absorption | ko04974 | 24 |
| Fat digestion and absorption | ko04975 | 2 |
| Bile secretion | ko04976 | 5 |
| Vitamin digestion and absorption | ko04977 | 1 |
| Mineral absorption | ko04978 | 9 |
| Alzheimer's disease | ko05010 | 60 |
| Parkinson's disease | ko05012 | 61 |
| Amyotrophic lateral sclerosis (ALS) | ko05014 | 4 |
| Huntington's disease | ko05016 | 56 |
| Prion diseases | ko05020 | 3 |
| Bacterial invasion of epithelial cells | ko05100 | 11 |
| Pathogenic Escherichia coli infection | ko05130 | 1 |
| Shigellosis | ko05131 | 2 |
| Leishmaniasis | ko05140 | 8 |
| Chagas disease (American trypanosomiasis) | ko05142 | 25 |
| African trypanosomiasis | ko05143 | 12 |
| Malaria | ko05144 | 3 |
| Toxoplasmosis | ko05145 | 14 |
| Amoebiasis | ko05146 | 45 |
| Staphylococcus aureus infection | ko05150 | 5 |
| Hepatitis C | ko05160 | 16 |
| Pathways in cancer | ko05200 | 44 |
| Colorectal cancer | ko05210 | 17 |
| Renal cell carcinoma | ko05211 | 12 |
| Pancreatic cancer | ko05212 | 16 |
| Endometrial cancer | ko05213 | 11 |
| Glioma | ko05214 | 15 |
| Prostate cancer | ko05215 | 19 |
| Thyroid cancer | ko05216 | 5 |
| Basal cell carcinoma | ko05217 | 9 |
| Melanoma | ko05218 | 6 |
| Bladder cancer | ko05219 | 4 |
| Chronic myeloid leukemia | ko05220 | 18 |
| Acute myeloid leukemia | ko05221 | 12 |
| Small cell lung cancer | ko05222 | 12 |
| Non-small cell lung cancer | ko05223 | 7 |
| Asthma | ko05310 | 2 |
| Autoimmune thyroid disease | ko05320 | 4 |
| Systemic lupus erythematosus | ko05322 | 11 |
| Rheumatoid arthritis | ko05323 | 3 |
| Allograft rejection | ko05330 | 4 |
| Graft-versus-host disease | ko05332 | 3 |
| Primary immunodeficiency | ko05340 | 5 |
| Hypertrophic cardiomyopathy (HCM) | ko05410 | 21 |
| Arrhythmogenic right ventricular cardiomyopathy (ARVC) | ko05412 | 14 |
| Dilated cardiomyopathy | ko05414 | 22 |
| Viral myocarditis | ko05416 | 14 |
